# Sex Chromosome-Specific Regulation in the *Drosophila* Male Germline But Little Evidence for Chromosomal Dosage Compensation or Meiotic Inactivation

**DOI:** 10.1371/journal.pbio.1001126

**Published:** 2011-08-16

**Authors:** Colin D. Meiklejohn, Emily L. Landeen, Jodi M. Cook, Sarah B. Kingan, Daven C. Presgraves

**Affiliations:** Department of Biology, University of Rochester, Rochester, New York, United States of America; University of California, Berkeley, United States of America

## Abstract

The evolution of heteromorphic sex chromosomes (e.g., *XY* in males or *ZW* in females) has repeatedly elicited the evolution of two kinds of chromosome-specific regulation: dosage compensation—the equalization of *X* chromosome gene expression in males and females— and meiotic sex chromosome inactivation (MSCI)—the transcriptional silencing and heterochromatinization of the *X* during meiosis in the male (or *Z* in the female) germline. How the *X* chromosome is regulated in the *Drosophila melanogaster* male germline is unclear. Here we report three new findings concerning gene expression from the *X* in *Drosophila* testes. First, *X* chromosome-wide dosage compensation appears to be absent from most of the *Drosophila* male germline. Second, microarray analysis provides no evidence for *X* chromosome-specific inactivation during meiosis. Third, we confirm the previous discovery that the expression of transgene reporters driven by autosomal spermatogenesis-specific promoters is strongly reduced when inserted on the *X* chromosome versus the autosomes; but we show that this chromosomal difference in expression is established in premeiotic cells and persists in meiotic cells. The magnitude of the *X*-autosome difference in transgene expression cannot be explained by the absence of dosage compensation, suggesting that a previously unrecognized mechanism limits expression from the *X* during spermatogenesis in *Drosophila*. These findings help to resolve several previously conflicting reports and have implications for patterns of genome evolution and speciation in *Drosophila*.

## Abstract 2

Suppression of X-linked transgene reporters versus normal expression of endogenous X-linked genes suggest a novel form of X chromosome-specific regulation in Drosophila testes, instead of sex chromosome dosage compensation or meiotic inactivation.

## Author Summary

Many species have heteromorphic sex chromosomes (*XY* males or Z*W* females) where one sex chromosome (the *Y* or *W*) has degenerated. In the somatic cells of mammals, worms, and flies, the *X*-to-autosome ratio of expression is equalized between the sexes by dedicated sex chromosome-specific dosage compensation systems. In the germline cells of male mammals and worms, however, the *X* chromosome is transcriptionally silenced early in meiosis. Here we have analyzed gene expression in *Drosophila* testes and show that the *X* chromosome lacks both of these types of chromosomal regulation. We find that *X* chromosome-wide dosage compensation is absent from most cells in the *Drosophila* male germline, and there is little or no evidence for *X* chromosome-specific inactivation during meiosis. However, another kind of sex-chromosome-specific regulation occurs. Testes-specific transgene reporters show much weaker expression when inserted on the *X* chromosome versus the autosomes, suggesting that some other, uncharacterized mechanism limits their expression from the *X* during spermatogenesis. The strong suppression of *X*-linked transgenes—but not *X*-linked endogenous genes—suggests that endogenous *X*-linked testes-specific promoters might have adapted to a suppressive *X* chromosome environment in the *Drosophila* male germline.

Heteromorphic sex chromosome systems (with *XY* males or *ZW* females) have evolved independently many times in animals and plants [1]. The difference between the sexes in chromosome copy number—e.g., two *X*'s in females but only one *X* in males—and the general absence of recombination between *X* and *Y* chromosomes have resulted in the evolution of sex chromosome-specific content and organization [2]–[4], rates of mutation and substitution [5], and most conspicuously, chromosome-level regulation. Two kinds of chromosomal regulation, in particular, have evolved repeatedly: dosage compensation, the process that equalizes *X* chromosome gene expression levels between the *XY* and *XX* sexes, and meiotic sex chromosome inactivation (MSCI), the facultative heterochromatinization and early transcriptional silencing of the *X* and the *Y* chromosome in germline cells entering meiosis in *XY* individuals [6],[7].

Dosage compensation, by far the better characterized of the two processes, has evolved in *XY* (mammals, *Drosophila*), *XO* (nematodes), but not, it seems, in *ZW* taxa (birds and Lepidoptera [8],[9]). While mechanisms of dosage compensation differ [10]—from silencing of a single *X* in *XX* female cells in eutherian mammals [11] to hypertranscription of the single *X* in *XY* males in *Drosophila* [12]—its function is to equalize the balance of *X* to autosomal gene expression in the two sexes [13]. Dosage compensation seems especially necessary for genes requiring similar expression in the two sexes, e.g., “housekeeping” genes [14], but perhaps less so for sex-specific ones. In the mouse female germline, dosage compensation appears mostly absent, as both *X* chromosomes are transcriptionally active in meiotic oocytes [15]. In the *Drosophila* male germline, the status of *X* chromosome dosage compensation is less clear. In male somatic tissues, the canonical dosage compensation complex (DCC), which comprises at least five proteins (MSL1, MSL2, MSL3, MLE, and MOF) and two RNAs (*roX1* and *roX2*), is targeted to degenerate high-affinity binding sequences enriched on the *X* chromosome and spreads to transcriptionally active genes where it facilitates hyper-transcription by directing acetylation of histone H4 on lysine 16 (H4Ac16) and enhancing the elongation of RNA polymerase II [10],[16],[17]. In the male germline, however, three of the five DCC proteins are not detectable, and H4Ac16 is not enriched on the *X* chromosome [18]. Two of the three DCC proteins that are absent in the testes have also been shown to be genetically dispensable for male fertility [18]–[21]. While MLE is present in testes and essential for male fertility, it does not localize to the *X* chromosome [18]–[21]. Microarray studies have nevertheless reported that the *X:* autosome of gene expression is equal in ovaries and testes, consistent with *X* chromosome dosage compensation [2],[3],[22]. Together these findings have suggested that a DCC-independent mechanism of *X* chromosome dosage compensation occurs in the *Drosophila* male germline [22],[23].

MSCI, which is less well characterized, occurs in mammals, nematodes, grasshoppers (*XO*), and possibly in birds [24]. In mice, MSCI is observable cytologically in pachytene spermatocytes as the *X* and *Y* chromosomes are sequestered into a distinct region of the nucleus [25]. During MSCI, multiple epigenetic modifications are localized to the *X* and *Y* (reviewed in [7]) and there are profound consequences for *X* chromosome gene expression—over 80% of *X-*linked genes decrease in expression by 10-fold or more [26]. Although 33 multicopy *X*-linked gene families are actively transcribed post-meiotically [27], most single-copy *X* chromosomal genes remain repressed in post-meiotic spermatids [26]. The function of MSCI is also less obvious than dosage compensation. The most general model posits that MSCI functions to silence selfish segregation distorter elements, which tend to accumulate preferentially on the *X* chromosome [28]–[32] (for other possible functions, see [7],[33]). Surprisingly, the existence of MSCI in *Drosophila* has been disputed for decades. Lifschytz and Lindsley argued that MSCI is universal and essential in all male *XY* taxa [6],[34]. They inferred MSCI in *Drosophila* from cytological and genetic findings including, but not limited to, their claim of allocyclic condensation of the *X* chromosome in primary spermatocytes and the dominant male-specific sterility of ∼75% of *X-*autosome translocations [6]. Consistent with Lifschytz and Lindsley's observations, Rastelli & Kuroda [18] found that H4Ac12, a histone mark enriched in heterochromatin in somatic cells, may label the *X-Y* cluster in late primary spermatocytes, whereas H3K4me3, a histone mark associated with active transcription, may be depleted from the *X-Y* cluster [35]. Kremer et al. [36], however, claim that the euchromatin of the *X* is entirely decondensed during a considerable period of first meiotic prophase, “contradictory to the results and the model of Lifschytz and Lindsley” (p. 158). McKee and Handel [33] further suggest that the cytological evidence for MSCI in *Drosophila* is inconclusive and the genetic data indirect. Instead, they argue that MSCI functions to prevent harmful crossing over between *X* and *Y* chromosomes in the *XY* sex, and as *Drosophila* male meiosis is achiasmate, MSCI need not occur.

Two recent experiments appear to provide renewed support for MSCI in *Drosophila*. First, Parsch and colleagues [37],[38] found that the promoter sequence of *ocnus*, an autosomal gene that encodes a putative sperm-specific histone (possibly a transition protein or protamine) [39], can drive strong testis-specific expression of a *lacZ* reporter when transgenes are inserted onto autosomes but not when inserted onto the *X* chromosome. Similar results have been observed for autosomal versus *X*-linked transgene inserts with the promoter of another testis-specific gene, *β2*-*tubulin* [40]. Second, using stage-specific microarray analyses of premeiotic, meiotic, and postmeiotic cell populations dissected from testes, Vibranovski et al. [41] found a small but significant excess of genes on the *X* chromosome that show reduced expression in meiotic relative to premeiotic stages of spermatogenesis. These studies are consistent with MSCI but provide somewhat conflicting pictures of the process. The transgene reporter assays, for instance, suggest that MSCI reduces expression from the *X* chromosome more than 5-fold [37],[40], whereas the microarray analyses suggest that MSCI is relatively weak, causing only ∼10% reduction in the expression of *X*-linked genes in meiotic cells on average [41].

In this article, we study the regulation of the *Drosophila X* chromosome in the male germline, revisiting earlier studies and reporting results from new analyses and experiments. First, we show that, contrary to previous reports, the *X* does not appear to undergo *X* chromosome dosage compensation in the *Drosophila* male germline. Second, we find no evidence for an excess of *X*-linked genes showing reduced expression in meiotic cells in the previously published microarray data [41], suggesting that MSCI in *Drosophila* either does not exist or is sufficiently weak to escape detection by microarray analysis. Finally, we find that the sperm-specific *ocnus* transgenes show much lower expression when *X*-linked versus autosomal, as previously reported [37],[38], but that this marked chromosomal difference is established early, in premeiotic cells. In the *Drosophila* male germline, then, both a lack of dosage compensation and an as yet unrecognized premeiotic mechanism appear to limit expression from the *X* chromosome. Our results help to resolve several seemingly conflicting findings regarding the regulation of the *X* chromosome in the *Drosophila* male germline and have implications for patterns of genome evolution and speciation in *Drosophila*.

## Results

### No *X* Chromosome Dosage Compensation in *Drosophila* Spermatocytes

**Figure 1:**
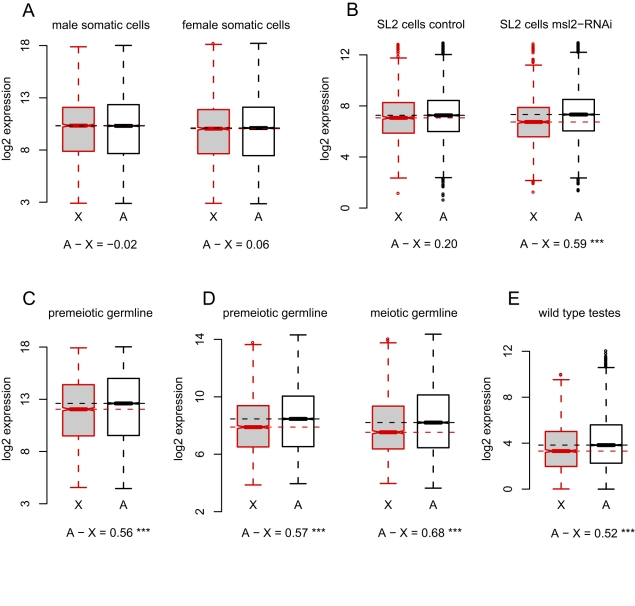
*X* chromosome and autosomal gene expression are consistent with no dosage compensation in *Drosophila* primary spermatocytes. (A) Autosome and *X* chromosome expression in cells in the male thorax and female thorax. (B) Autosome and *X* chromosome gene expression from control cells and from *SL2* cells in which dosage compensation has been knocked down by RNAi against *msl2* [43]. (C–E) Autosome and *X* chromosome gene expression in the male germline. Premeiotic cells were dissected from the apical tip of the testes; meiotic cells were dissected from the proximal region of the testes. Data in (C) are from Agilent *Drosophila* gene expression microarrays; (D) shows previously published data [41] using Affymetrix GeneChips. (E) Previously published [48] RNA-seq data from wild-type testes. *** *p*<0.001 (Mann-Whitney test).

In the *Drosophila* male germline, decreased expression from the *X* chromosome could plausibly reflect MSCI or the stage-specific loss of *X* chromosome dosage compensation. To distinguish these possibilities, we asked if *X* chromosome dosage compensation occurs in premeiotic spermatocytes. As controls, we first estimated levels of *X* chromosome dosage compensation in male somatic tissues, using microarrays to assay gene expression in thorax dissected from adult males and females. Cells in the thorax are likely to be similar between the sexes (i.e., largely comprising flight muscle), thus minimizing the confounding effects of sex-specific gene expression. Global gene expression is indeed highly correlated between male and female thorax (*r = *0.972, *p*<10^−15^; Figure S1). Furthermore, the difference in median expression level between *X*-linked and autosomal probes is negligible, with autosomal probes showing 0.98- and 1.04-fold higher expression levels in males and females, respectively, than *X*-linked ones (Figure 1A). As expected, in these cells, the *X* chromosome is fully dosage compensated and there is equal expression from the *X* and the autosomes in both sexes.

To determine the magnitude of the *X*-autosome difference in expression expected in the absence of dosage compensation, we referred to data from published microarray experiments using *Drosophila* male-like *SL2* cells in which mRNA encoding the limiting dosage compensation protein, MSL2 [42], was knocked down by RNA interference (RNAi) [43]. In control cells treated with RNAi against GFP, autosomal genes have a slight (1.15-fold) but significantly higher median expression than *X*-linked genes (Mann-Whitney *P*_MW_ = 0.01; Figure 1B), whereas in *msl2*-knockdown cells, autosomal genes have a 1.51-fold higher median expression than *X*-linked ones (*P*_MW_<10^−15^; Figure 1B). Impairment of the DCC in these experiments therefore results in a 1.31-fold reduction in *X-*linked gene expression relative to the autosomes. Similar RNAi knockdown of *msl2* and *mof* in *SL2* cells, with gene expression measured by RNA-seq, results in a 1.35-fold decrease in *X*-linked gene expression relative to autosomes [44]. Similarly, male larvae carrying mutations at the *roX* loci show a 1.20-fold difference between *X* and autosomal expression [45],[46]. Loss of DCC-dependent dosage compensation therefore results in a 1.2- to 1.4-fold decrease in expression of *X*-linked genes compared to autosomal ones.

To directly test for *X* chromosome dosage compensation in the *Drosophila* male germline, we used microarrays to assay gene expression in cells dissected from the apical tip of the testes with the somatic and DCC-expressing [18] cells of the surrounding testes sheath removed. These apical dissections comprise hub cells, germline and somatic stem cells, somatic cyst cells, mitotic spermatogonia, and early primary spermatocytes, which grow for approximately 3 d following their last mitotic division prior to the first meiotic division [47]. We chose these dissected cells (for convenience, hereafter called “premeiotic”) rather than whole testes to avoid conflating our results with meiosis-specific *X* chromosome regulation, such as MSCI. In these premeiotic cells, median absolute expression of autosomal probes is 1.47-fold higher than *X*-linked probes (*P*_MW_<10^−6^; Figure 1C). The precise magnitude of this *X-*autosome difference depends somewhat on the extent to which lowly expressed probes are filtered from the analysis but ranges from 1.39-fold to 1.54-fold (Figure S2).

To evaluate the generality of our estimated ∼1.5-fold difference in *X*-autosome expression, we analyzed data from two previous studies. In the first study, Vibranovski et al. [41] dissected three populations of cells from *Drosophila* testes: apical tips enriched for premeiotic cells; proximal cells enriched for late-stage primary and meiotically dividing spermatocytes (hereafter “meiotic”); and distal cells enriched for postmeiotic cysts and elongating spermatids (hereafter “postmeiotic”). We observe a similar *X*-autosome expression difference in their premeiotic dissections that included the somatic testis sheath [41]: autosomal probes show 1.48-fold higher median expression than the *X* (*P*_MW_<10^−10^; Figure 1D). In proximal dissections (which did *not* include testis sheath) [41], the autosomes show a 1.60-fold higher median expression than the *X* (*P*_MW_<10^−10^; Figure 1D). In the second study, Gan et al. generated RNA-seq data from whole testes [48]. Based on 19,849,063 uniquely mapped reads, we estimate that autosomal genes show 1.44-fold greater expression versus *X-*linked genes (*P*_MW_<10^−10^; Figure 1E).

**Figure 2:**
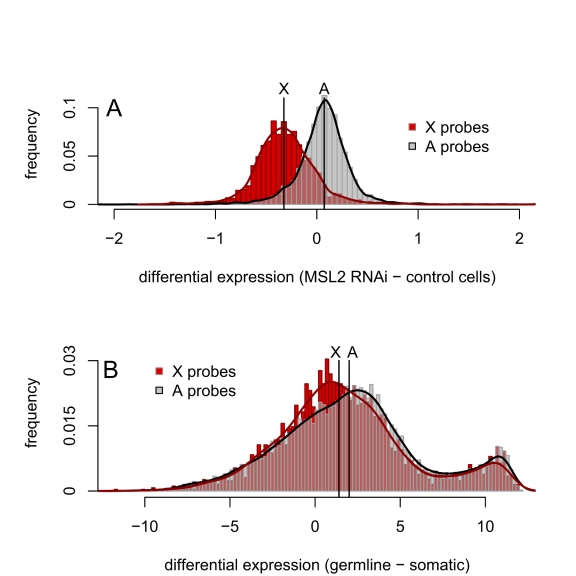
Differences in *X*-linked and autosomal gene expression between male-like *SL2* cells with and without dosage compensation are similar to the differences between somatic and germline cells in males. (A) The distributions of expression differences between *msl2*-RNAi and control cells for *X* chromosome and autosomal probes [43]. (B) The distributions of expression differences between male germline cells and male thorax tissue for *X* and autosomal probes. Black lines indicate the median values of each distribution; the difference between the median log2 expression of autosomal and *X-*linked probes is 0.398 in (A) and 0.568 in (B).

In addition to comparing expression from the *X* and the autosomes within a tissue, we compared differences in expression between cell types for *X-*linked and autosomal genes. The *msl2*-RNAi experiments [43] again provide a useful control, where the median difference in expression between *msl2*-knockdown cells and control cells is 1.05-fold for autosomal probes and 0.80-fold for *X*-linked probes (Figure 2A). The difference in expression levels between cells with and without dosage compensation in these experiments is therefore 1.32-fold lower for *X-*linked genes than for autosomal genes. The analogous difference in expression between germline and somatic cells is complicated by large tissue-specific differences in gene expression (Figure 2B). However, despite the confounding effects of tissue-specific expression, the difference in median expression levels between male thorax and premeiotic dissections is 1.48-fold lower for *X*-linked probes than for autosomal probes, a value similar to that from the *msl2*-RNAi experiments. Thus, across three independent experiments using differently dissected stages of spermatogenesis, whole testes, and across three different gene expression assays (Affymetrix microarrays, Agilent microarrays, and RNA-seq), we find that the *X* chromosome has reduced expression relative to the autosomes. The magnitude of this difference is strikingly similar to that seen for experimentally manipulated cells lacking dosage compensation. We therefore conclude that *X* chromosome dosage compensation is absent from most of the *Drosophila* male germline.

### *X* Chromosome Expression in the *Drosophila* Female Germline and in Germline Stem Cells

**Figure 3:**
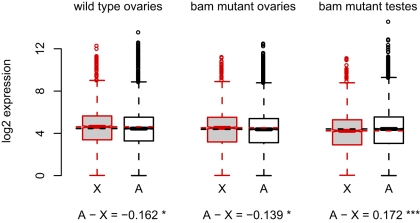
*X* chromosome and autosome expression is similar in ovaries and germline stem cells. RNA-seq data [48] from wild-type ovaries, *bam* mutant ovaries, and *bam* mutant testes. * *p*<0.05, *** *p*<0.001 (Mann-Whitney test).

To test if reduced expression from the *X* is a general feature of germline expression, rather than a male-specific absence of germline *X* chromosome dosage compensation, we estimated *X* and autosomal expression levels in wildtype ovaries from the RNA-seq data of Gan et al. [48]. In contrast to the testes, autosomal genes show 0.89-fold *lower* median expression than *X-*linked genes (*P*_MW_ = 0.027; Figure 3). Reduced expression from the *X* relative to the autosomes is therefore specific to the testes and not a general property of germline gene expression in *Drosophila*.

We also estimated *X* and autosomal expression levels using RNA-seq data from mutant male and female germline tissue in which development is arrested at an early stage [48]. The *bag-of-marbles (bam)* gene is required for male germline cells to exit the mitotic divisions and begin primary spermatocyte development, and *bam* mutant gonads are consequently enriched for undifferentiated germ-line stem cells and mitotic spermatogonia [49],[50]. In *bam* ovaries, *X-*linked and autosomal expression levels are similar to wild-type ovaries: autosomal genes show 0.91-fold lower median expression than *X*-linked genes (*P*_MW_ = 0.035; Figure 3). In *bam* mutant testes, however, we find that autosomal genes show a 1.13-fold higher median expression than *X-*linked genes (Figure 3), a value that is significantly different from zero (*P*_MW_<0.001), but smaller than the ∼1.45-fold difference seen in wild-type testes. Notably, primary spermatocytes are absent from *bam* testes but likely constitute most of the premeiotic cells dissected from the apical tip of the testes. The discrepancy in the *X*-autosome difference in expression between *bam* testes (1.13-fold) and premeiotic dissections (1.45-fold) therefore suggests that the *X*-autosome difference in expression increases in differentiating primary spermatocytes.

### *X* Chromosome Expression in Late Meiotic Spermatocytes—A Modest Dearth of Upregulated Genes But No Excess of Downregulated Genes

The magnitude of the *X*-autosome difference in expression in *Drosophila* testes described above is consistent with a lack of *X* chromosome dosage compensation but not with global inactivation of the *X*. In mice, MSCI initiates at pachytene of prophase I [7], resulting in transcriptional silencing of more than 80% of *X*-linked genes [26],[27]. Assuming *Drosophila* males experience a similar stage-specific inactivation of the sex chromosomes, cells in late prophase I undoubtedly represent a small proportion even of meiotic dissections enriched for late primary spermatocytes. Any signal of MSCI might therefore only be detected by comparing the changes in *X* and autosomal expression across different stages of spermatogenesis [41]. As described above, Vibranovski et al. [41] dissected populations of cells from wild-type testes enriched for premeiotic, meiotic, and postmeiotic cells and assayed gene expression with microarrays. Using a novel Bayesian analysis of all *X-*linked and autosomal probes, these authors reported a small but significant excess of *X-*linked genes downregulated in meiotic dissections relative to premeiotic dissections (56% of *X-*linked versus 52% of autosomal genes identified as testis-expressed in FlyAtlas [51], see Figure 3 in [41]).

To assess the robustness of this putative signal of MSCI, we reanalyzed these microarray data by identifying individually significant changes in gene expression between stages of spermatogenesis with probe-level *t* tests, using a false discovery rate (FDR) of 0.05 to correct for multiple tests (see Figure S3) [52]. Our conclusions do not qualitatively change with increasing FDR stringency or when using an arbitrary 2-fold cutoff for determining significant changes in expression between stages of spermatogenesis (Tables S1–S6). Table 1 shows the number of probe sets significantly differentially expressed by chromosome arm between premeiotic, meiotic, and postmeiotic cells. In the early transition (premeiotic→meiotic cells), 38% and 37% of *X*-linked and autosomal probes, respectively, show significant decreases in expression, whereas 24% and 31% of *X*-linked and autosomal probes show significant increases in expression, respectively (Table 1). While the proportion of genes downregulated in meiotic cells is similar for the *X* and autosomes (Fisher's exact test *P*_FET_ = 0.190), the *X* has a significant paucity of genes upregulated in meiotic cells (*P*_FET_ = 4.5×10^−10^). Of those probes that show significant changes in the early transition, the median magnitude of decreased expression is similar for the *X* and autosomes (Table 2), but *X-*linked probes show significantly smaller increases in expression (*P*_MW_ = 3.66×10^−5^). The deficit of upregulated *X-*linked genes in the early transition was found by Vibranovski et al. [41], but they also reported a small but significant excess of *X-*linked genes downregulated in the early transition, which we do not observe.

**Table 1:** Number of genes with significant differences in expression between stages of spermatogenesis.

Chr Arm	# Expressed	Early Changes (Premeiosis:Meiosis)		Late Changes (Meiosis:Postmeiosis)		Net Change (Premitosis:Postmeiosis)	
		Down	Up	Down	Up	Down	Up
2L	2,204	764 (34.7%)	739 (33.5%)	846 (38.4%)	672 (30.5%)	898 (40.7%)	879 (39.9%)
2R	2,356	926 (39.3%)	699 (29.7%)	862 (36.6%)	762 (32.3%)	947 (40.2%)	941 (39.9%)
3L	2,335	834 (35.7%)	741 (31.7%)	822 (35.2%)	714 (30.6%)	934 (40.0%)	937 (40.1%)
3R	3,009	1,096 (36.4%)	903 (30.0%)	1,094 (36.4%)	911 (30.3%)	1,237 (41.1%)	1,185 (39.4%)
4	58	39 (67.2%)	6 (10.3%)	22 (37.9%)	19 (32.8%)	29 (50.0%)	14 (24.1%)
X	1,943	741 (38.1%)	**469 (24.1%)**	**601 (30.9%)**	656 (33.8%)	744 (38.3%)	734 (37.8%)
A^a^	9,904	3,620 (36.6%)	**3,082 (31.1%)**	**3,624 (36.6%)**	3,059 (30.9%)	4,016 (40.6%)	3,942 (39.8%)
X versus A (*FET p*-value)		0.190	**4.51**×**10^−10^**	**1.64**×**10^−6^**	0.013	0.065	0.099

a Autosomal totals exclude genes on the 4th chromosome.

**Table 2:** Median log2 magnitude of changes in expression between stages of spermatogenesis.

Chr Arm	Early Changes (Premeiosis:Meiosis)			Late Changes (Meiosis:Postmeiosis)			Net Change (Premeiosis:Postmeiosis)		
	Down	Up	Up + Down	Down	Up	Up + Down	Down	Up	Up + Down
2L	−0.86	0.96	0.10	−1.34	0.88	−0.46	−1.71	1.07	−0.64
2R	−0.86	1.01	0.15	−1.32	0.93	−0.39	−1.73	1.07	−0.66
3L	−0.86	0.92	0.06	−1.38	0.81	−0.57	−1.64	0.94	−0.70
3R	−0.85	0.85	0.00	−1.32	0.88	−0.44	−1.68	0.99	−0.69
4	−0.87	1.11	0.24	−1.03	1.49	0.46	−1.46	0.81	−0.65
X	−0.91	**0.84**	−0.07	**−1.15**	0.93	−0.22	**−1.45**	**0.89**	−0.56
A^a^	−0.86	**0.92**	0.06	**−1.33**	0.88	−0.45	**−1.69**	**1.01**	−0.68
X vs A (*MW P*-value)	0.143	**3.66**×**10^−5^**	—	**1.28**×**10^−4^**	0.891	—	**4.97**×**10^−6^**	**6.84**×**10^−4^**	—

a Autosomal totals exclude genes on the 4th chromosome.

A different pattern emerges for the late transition (meiotic→postmeiotic cells): 31% and 37% of *X*-linked and autosomal probes, respectively, show significant decreases in expression, whereas 34% and 31% of *X*-linked and autosomal probes show significant increases in expression, respectively (Table 1). The *X* has a significant deficit of probes downregulated in postmeiotic cells (*P*_FET_ = 1.6×10^−6^), and a marginally significant excess of probes upregulated in postmeiotic cells (*P*_FET_ = 0.013). During the late transition, the magnitude of decreased expression is significantly less for the *X* than for the autosomes (*P*_MW_ = 1.28×10^−4^; Table 2), whereas the magnitude of increased expression is similar (*P*_MW_ = 0.891).

The behavior of the *X* chromosome in the *Drosophila* male germline is therefore distinct from MSCI as it occurs in mammals [26], at least at the resolution afforded by these dissections. Instead of an inactivation of the *X* chromosome during prophase I that results in strong decreases in the number and magnitude of expressed *X*-linked genes that then largely persists throughout the remainder of spermatogenesis [26],[27], we see an overall dampening of the change in gene expression on the *X* relative to the autosomes: a smaller proportion of *X*-linked genes change in expression at either stage of spermatogenesis and, of those that do change, the median fold-change is ∼10%–20% smaller than that seen on the autosomes (Tables 1 and 2, Figure S3).

In contrast to the rest of the genome, the largely heterochromatic fourth chromosome shows an excess of downregulated genes in the meiotic dissections (Table 1): 67% of fourth chromosome probes decrease expression in the early transition (Fisher's exact test of fourth chromosome probes versus all others: *P*_FET_ = 3.5×10^−6^), whereas only 10% increase expression (*P*_FET_ = 7.6×10^−4^). The magnitude of expression changes at both transitions is, however, similar for the fourth and the *X* and autosomes (Table 2). The fourth chromosome results show that combining these testes dissections with microarray analysis [41] provides sufficient resolution to detect large-scale chromosome-wide changes in expression during spermatogenesis. The absence of a comparable pattern for the *X* chromosome is thus not simply due to a lack of statistical power or experimental resolution.

It is worth noting that the genes showing significant changes in expression in meiotic cells relative to premeiotic ones fit what might be expected of *Drosophila* spermatogenesis. Those showing significantly elevated expression in meiotic cells, for instance, are enriched for functions in microtubule activity (e.g., dynein complex, axoneme function) and sperm development (e.g., vesicle and membrane docking), whereas those showing significantly reduced expression are enriched for transcriptional functions (e.g., RNA pol II activity, RNA splicing, mRNA processing). These findings are consistent with overall reduced postmeiotic *de novo* transcriptional activity and a shift to posttranscriptional mechanisms of development during spermatogenesis in *Drosophila* [47],[49],[53],[54].

### Differential Somatic Contamination between Premeiotic and Meiotic Cell Dissections

Our inference that there is little signal of MSCI in these dissections [41] is conservative, as the proportion of *X*-linked genes downregulated in meiotic cells is likely overestimated in these microarray data. The premeiotic dissections from the apical tip of the testes included the surrounding testes sheaths—which are somatic, express the DCC [18], and are thus presumably dosage compensated—whereas the meiotic dissections from the proximal regions of the testes included only germline cells [41] that lack *X* chromosome dosage compensation (see above). The presence of contaminating sheath tissue could therefore inflate *X* chromosome expression levels in premeiotic samples, causing a spurious inference of downregulation in meiotic cells. To test for an effect of the presence of somatic sheath cells on the observed expression of *X*-linked genes in premeiotic versus meiotic cells in the microarray data, we dissected three cell populations from the testis: apical tips with testis sheath (premeiotic + sheath), apical tips without testis sheath (premeiotic), and proximal dissections without sheath (meiotic). Using quantitative reverse transcriptase-PCR (qPCR), we assayed expression of 15 genes: 12 at different cytological positions on the *X* with high overall expression levels in the microarray data and significant changes from meiotic cells to premeiotic cells, and three chosen as normalizing controls because they showed no significant change in expression between premeiotic and meiotic cells (*CG1440* on the *X*, *Tub84D* on *3R*, and *CG10252* on *3R*; see Materials and Methods).

**Figure 4:**
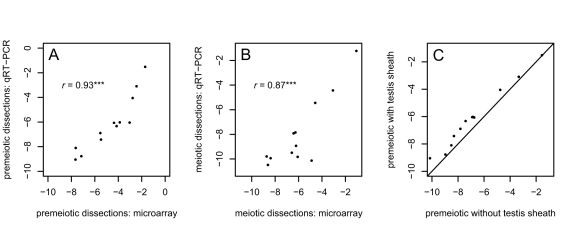
qRT-PCR analysis indicates the contaminating effect of testis sheath has a detectable effect on gene expression. (A & B) qRT-PCR results for 12 genes from premeiotic and meiotic dissections show good correspondence with previously published microarray results [41]. (C) Apical dissections (premeiotic cells) including the testis sheath show slight but detectable increases in the expression of *X*-linked genes relative to apical dissections from which the sheath has been removed. *** *p*<0.001.

Relative expression levels of all 12 genes in both our premeiotic dissections including sheath and meiotic dissections recapitulate the previous microarray analysis well (Figure 4A,B) [41]. For 11 of the 12 genes, our qPCR results show the same direction and similar magnitude of expression change between stages (Table 3). All 12 *X*-linked genes show greater expression in premeiotic cells with the testis sheaths than when the sheaths are removed (binomial test *p* = 4.9×10^−4^; Figure 4C). This difference is significant for six of the 12 genes (*p*<0.05), and highly significant when all 12 genes are pooled (*p*<10^−8^; Table 3). These results suggest that, on average, the differences in expression levels of *X*-linked genes between premeiotic and meiotic cells in Vibranovski et al. [41] might be overestimated by as much as 36%. Consequently, the proportions and magnitudes of *X*-linked genes upregulated and downregulated in meiosis shown in Tables 1 and 2 are likely underestimates and overestimates, respectively. Sheath contamination also likely contributes to the greater difference between *X* and autosomal expression seen in the meiotic dissections relative to the premeiotic dissections (Figure 1C).

**Table 3:** Contamination by somatic testis sheath has detectable effects on changes in gene expression between stages of spermatogenesis.

Gene	Cytological Location	*X* Chromosome Coordinate	Ps - M^a^ Microarray	Ps - M^a^ qRT-PCR	Ps - Pn^b^ qRT-PCR	Sheath Effect^c^	Sheath Effect *p* Value^d^
*CG14629*	1E	945569	0.72	1.02	0.91	89%	0.088
*CG3655*	1E	967938	0.92	1.69	0.40	24%	0.149
*CG14805*	2B	1771351	2.03	3.80	0.81	21%	0.044
*CG14806*	2B	1774329	0.45	1.34	0.23	17%	0.032
*Notch*	3C	3028904	1.29	1.71	0.18	11%	0.324
*dunce*	3C	3070474	0.93	2.07	0.88	43%	0.036
*Cdc42*	18E	19591116	1.63	1.39	0.70	50%	0.011
*CG12703*	18E	19644832	1.73	1.77	0.68	38%	0.047
*Cyp6v1*	19E	20528810	0.56	0.89	1.10	124%	0.113
*CG1835*	19E	20539348	−0.81	−0.29	0.03	−9%	0.544
*penguin*	19E	21217529	1.68	4.09	0.86	21%	0.055
*Helicase*	20A	21256541	1.90	2.60	1.08	42%	0.014
All genes			1.09	1.84	0.66	36%	6.68×10^−9^

Gene expression differences are log2 fold-change between the various dissections. qRT-PCR values were normalized by three control genes (see Materials and Methods).

a Premeiotic dissections with testis sheath included − meiotic dissections.

b Premeiotic dissections with testis sheath included − premeiotic dissections with testis sheath removed.

c Sheath effect is calculated as the ratio of (Ps−Pn)/(Ps−M) from qRT-PCR.

d *p-*value calculated from paired *t* tests between Ps and Pn.

### *X* Chromosome-Specific Reduction in *WOL* and *YLZ* Transgene Expression Is Independent of Spermatogenic Stage

We next extended the analysis of two transgenes used by Hense et al. as putative reporters of MSCI in *Drosophila* [37]. In both transgene constructs, *lacZ* expression is driven by a 110-bp promoter-containing sequence from the 5′-region of *ocnus* (*ocn*), an autosomal (*3R*) gene that encodes a putative sperm-specific histone [37],[39]: *P[wFl:ocn:lacZ:w^+^]* and *P[y^+^:YEStes:ocn:lacZ]* (hereafter *WOL* and *YLZ*, respectively). *WOL* and *YLZ* constructs differ from one another in two ways: *YLZ* possesses the *ocn* 3′-UTR downstream of *lacZ* as well as flanking Suppressor of Hairy-wing binding sites, which function as chromosomal insulators [37]. Previously, Hense et al. [37] showed that *X*-linked inserts of the *WOL* and *YLZ* transgenes show significantly lower expression than autosomal inserts in both mRNA and protein levels in males.

**Figure 5:**
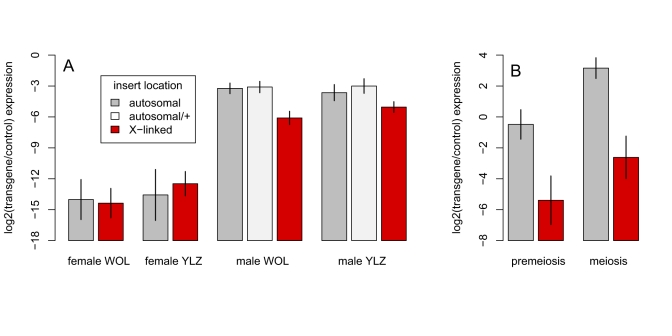
Sex, chromosome, and spermatogenic stage effects on the expression of *WOL* and *YLZ* transgenes. (A) Expression of *ocn:lacZ* transgenes is low or absent in females, and is significantly lower for *X*-linked inserts than autosomal inserts in males. Bars indicate the mean expression measured from 8 *X*-linked and 8 autosomal *WOL* inserts and 6 *X*-linked and 5 autosomal *YLZ* inserts. RNA was extracted from whole adult flies and expression from autosomal inserts was measured in both heterozygous and homozygous male and homozygous female genotypes. (B) The difference between *X-*linked and autosomal inserts persists from premeiotic to meiotic cells in the male germline. A subset of genotypes (two *X-*linked and one autosomal *WOL* and two *X-*linked and one autosomal *YLZ*) shown in panel A were used for dissections (see Materials and Methods for details). In both panels, gene expression is measured relative to a *Rpl32* control probe and error bars indicate 95% confidence intervals.

We confirmed that the transgenes show strong sex- and chromosome-specific expression differences by assaying mRNA transcript levels in whole adult females homozygous for single *X*-linked or autosomal transgene inserts and in whole adult males hemizygous for single *X*-linked inserts and heterozygous and homozygous for autosomal inserts. Our qPCR results show, as reported by Hense et al. [37], that *lacZ* expression from both transgenes is much higher in males than in females (Table 4; Figure 5A), consistent with the testis-specific function of *ocn*. We also find a highly significant interaction between sex and chromosomal location (Table 4): *X*-linked inserts show ∼5-fold lower *lacZ* expression than autosomal inserts in males but not in females. The reduced *lacZ* expression from *X*-linked transgenes is thus specific to males.

**Table 4:** Sex, transgene, and chromosomal effects on the expression of *ocn* transgenes.

Source of Variation	SumSq	*df*	*F*	*p* Value
1. Sex (male versus female)	1,626.18	1	1,309.67	<1×10^−15^
2. Location (*X* versus A/A versus A/+)	54	2	21.74	1.73×10^−8^
3. Transgene (*WOL* versus *YLZ*)	7.31	1	5.89	0.0172
4. Sex × location	25.9	2	10.43	0.0001
5. Sex × transgene	1.28	1	1.03	0.3129
6. Location × transgene	6.59	2	2.65	0.0758
7. Sex × location × transgene	2.42	2	0.97	0.3819
Residuals	116.72	94		

To investigate stage-specific expression of *WOL* and *YLZ* transgenes in testes, we assayed reporter expression in premeiotic and meiotic cells dissected from testes with the somatic sheath removed. If the difference between *X*-linked and autosomal transgene insertions is due to transcriptional silencing of the *X* in spermatocytes during meiosis, as expected under MSCI, then *lacZ* expression from *X*-linked but not autosomal inserts should be strongly reduced in meiotic versus premeiotic dissections. However, *X-*linked *WOL* and *YLZ* transgenes show no *stage-specific* repression in the *Drosophila* male germline. First, *X*-linked inserts show much lower (∼30-fold) *lacZ* expression than autosomal inserts in both premeiotic and meiotic cells (Figure 5B; Table 5, line 1). Second, relative to the control gene *RpL32*, *lacZ* expression from both transgenes is significantly higher in meiotic cells versus premeiotic cells (Figure 5B; Table 5, line 2); this increase is likely due to reduced transcript abundance of *RpL32* in meiotic dissections (unpublished data). However, there is no significant interaction between stage of spermatogenesis (premeiotic versus meiotic) and chromosomal location (*X* versus autosome; Table 5, line 4): both autosomal *and X*-linked transgenes show similarly increased relative expression in meiotic cells (Figure 5B).

**Table 5:** Spermatogenic stage and chromosomal effects on the expression of *ocn* transgenes.

Source of Variation	SumSq	*df*	*F*	*p* Value
1. Location (*X* versus autosomes)	380.54	1	54.001	1.38×10^−9^
2. Stage (premeiotic versus meiotic)	140.98	1	20.006	4.22×10^−5^
3. Transgene (*WOL* versus *YLZ)*	6.28	1	0.891	0.350
4. Stage × location	2.52	1	0.358	0.552
5. Stage × transgene	6.5	1	0.922	0.341
6. Location × transgene	1.58	1	0.224	0.638
7. Stage × location × transgene	0.17	1	0.024	0.877
Residuals	366.44	52		

These findings show that the *WOL* and *YLZ* transgene inserts on the *X* chromosome have much lower expression than autosomal inserts in premeiotic cells and that this chromosomal effect persists without significant change in meiotic cells. The overall lower expression of *X*-linked versus autosomal inserts reported by Hense et al. [37] cannot therefore be attributed to a meiosis I-specific inactivation of the *X* chromosome. Furthermore, the magnitude of lower expression of *X*-linked versus autosomal inserts—∼30-fold in premeiotic cells and ∼5-fold in whole males for hemizygous *X*-linked inserts versus heterozygous autosomal ones—is too large to be explained by a lack of dosage compensation (see Figure 5A and also [37]). The *WOL* and *YLZ* transgenes thus appear to reveal a previously uncharacterized mechanism of reduced expression from the *X* chromosome, distinct from the lack of dosage compensation and distinct from mammal-like MSCI, that is established early in cells of the *Drosophila* male germline and persists at least into meiosis.

## Discussion

The findings reported here lead to several new conclusions regarding expression from the *X* chromosome in *Drosophila* testes. First, expression levels of genes on the *X* chromosome and the autosomes in *Drosophila* testes are not equal, contrary to previous reports [3],[22]. Instead, *X* chromosome dosage compensation appears to be absent in the *Drosophila* male germline, consistent with the absence of the DCC in the testes [18]. Second, we find no indication of a chromosome-wide, meiosis-specific silencing of gene expression from the *X* chromosome in data from microarrays or the *ocnus* transgenes. Although we cannot formally exclude that MSCI occurs in flies, the recent expression-based assays provide little evidence for it. Instead, we show that the markedly reduced expression driven by the autosomal *ocnus* promoter from *X*-linked versus autosomal transgenes is established in the testes well before meiosis I. Thus, expression from these *X*-linked transgenes is constrained throughout much of the *Drosophila* male germline by an uncharacterized mechanism, in a manner distinct from MSCI as it occurs in mammals [7].

### *X* Chromosome and Autosomal Expression of Endogenous Genes in the *Drosophila* Male Germline

Expression of endogenous *X*-linked genes in *Drosophila* testes was thought to be affected by two modes of chromosomal regulation: DCC-independent *X* chromosome dosage compensation was thought to equalize *X* and autosomal expression [3],[22], and MSCI was thought to cause reduced expression from the *X* in early meiosis [6],[37],[41]. A third possible cause of *X*-autosome differences in expression involves evolved differences in chromosomal gene content. We discuss all three of these possibilities below.

We have found that the *X* chromosome shows ∼1.5-fold significantly lower overall expression than the autosomes in premeiotic cells dissected from the apical tip of the testes in our microarray data, in those of Vibranovski et al. [41], and in RNA-seq data from whole testes [48]. The magnitude of these *X*-autosome differences is strikingly similar to that seen in cells in which DCC-mediated dosage compensation was experimentally impaired (Figure 1;[43],[44]), suggesting that *X* chromosome dosage compensation is absent in *Drosophila* testes. It is, however, important to distinguish *X* chromosome dosage compensation (like that mediated by the DCC) from other processes not specific to the *X* chromosome that ameliorate gene dose differences, sometimes termed buffering or (confusingly) dosage compensation [55]. Gene expression analyses, for instance, indicate that hemizygous autosomal genes in deficiency-bearing *Drosophila* adults have ∼1.5-fold lower expression than wildtype [56]. These experiments and dose-response analyses in aneuploid cells [44] show that 2-fold differences in gene dose are dampened by a buffering mechanism acting at the transcriptional level, resulting in only a ∼1.5-fold expression difference, on average. We speculate that this kind of buffering mitigates the 2-fold gene dose difference between *X* and autosomes in the male germline, resulting in a ∼1.5-fold *X*-autosome difference in expression. Thus, the simplest explanation for our observations—given the *X*-autosome difference in expression, the absence of the DCC or any known analogs, and the lack of H4Ac16 (or H4Ac5 and H4Ac8) enrichment on the *X* [18]—is that a dedicated *X* chromosome-wide mechanism of dosage compensation analogous to the somatic DCC is absent in the male germline. It is worth noting that while *X* chromosome dosage compensation is essential for male viability, male-like cells with compromised DCC-mediated dosage compensation are viable and show no reduction in doubling times [43]. *X* chromosome dosage compensation thus appears essential for somatic development but not cell viability or, we infer, germline function.

Lifschytz and Lindsley [6] inferred MSCI in *Drosophila* from two lines of evidence: the dominant chromosomal male-sterility of most *X*-autosome translocations and cytological observations. The translocation data are, however, indirect [33] and the original cytological data do not appear definitive [36]. The *ocnus* and *β2-tubulin* transgene experiments [37],[40] along with microarray analyses of staged testes dissections provided what seemed to be new and complementary functional evidence for MSCI in flies. As we have shown here, however, the reduced expression of *X*-linked transgenes in *Drosophila* testes does not reflect a meiosis-specific process, and microarray data fail to show evidence for overall reduced expression from the *X* chromosome in cells enriched for meiosis I-stage spermatocytes (Tables 1 and 2; see also Supporting Information). Thus, any cytological differences between the *X* and the autosomes in male meiosis do not seem to result in chromosome-wide silencing of gene expression. In support of this conclusion, a recently published study of gene expression in developing larval testes also failed to find evidence of MSCI in *Drosophila* [57].

There are at least three caveats to our conclusion that expression of endogenous genes provides little evidence for MSCI. First, two patterns in the microarray data might be construed as evidence of MSCI. While we detect no excess of *X-*linked genes downregulated in meiotic cells, there is a modest dearth of upregulated *X*-linked genes (Table 1); and when considering all probes on the microarrays, ignoring whether they show individually significant changes in expression, there is a significant difference between the median magnitude of change from premeiotic to meiotic dissections for *X-*linked (0.97-fold) and autosomal (1.02-fold) probes (*P*_MW_<10^−6^). These patterns may correspond to the effect detected by an earlier Bayesian analysis [41] and may reflect MSCI taking place in a small subset of spermatocytes in the meiotic cell dissections. However, we hesitate to take these subtle expression differences as evidence of MSCI. For one, a dearth of upregulated *X*-linked genes in meiotic cells, but no corresponding excess of downregulated *X*-linked genes, is not necessarily expected under MSCI. Furthermore, the weakly reduced magnitude of expression of *X*-linked genes in meiotic cells could be due to the confounding effects of the presence of DCC-compensated testis sheath tissue in the premeiotic dissections but not the meiotic ones (Figure 4, Table 3).

Second, expression-based assays may have limited power to detect MSCI in flies, for two technical reasons. First, while the stage-specific premeiotic and meiotic testes dissections are likely enriched for different cell populations—mitotic spermatogonia/premeiotic spermatocytes versus meiotic spermatocytes, respectively—other cell types and stages undoubtedly contaminate them [41]. Indeed, the strong signal of MSCI in mammal expression analyses, in which more than 80% of genes on the *X* show greater than 10-fold reduced expression in pachytene spermatocytes [26], could result from purer samples. Second, as microarrays measure transcript abundance and not transcription per se, they may not be ideal for measuring an abrupt, stage-specific reduction in gene expression. Even if transcription on the *X* were completely silenced in meiotic spermatocytes, transcripts produced earlier may persist—particularly during spermatogenesis—thus dampening any signal of MSCI. Transcript persistence does not, however, seem to suppress the signal of MSCI in mammals. Furthermore, the heterochromatic-dot fourth chromosome shows a robust excess of downregulated genes in the meiotic dissections (Table 1), suggesting that such an effect is detectable using the current microarray analyses and dissections. If there is an effect of MSCI on gene expression in the *Drosophila* germline, its signal must be weaker than that seen for the dot fourth chromosome and heterochromatic genes.

Third, there is, in addition to MSCI and the absence of *X* chromosome dosage compensation, another possible cause for the *X*-autosome difference in gene expression in *Drosophila* testes. Genes with male-biased expression (i.e., those expressed at higher levels in males than in females) are significantly underrepresented on the *Drosophila X* chromosome [2],[3]. This evolutionary “demasculinization” of the *X* has previously been attributed to the long-term accumulation of gene duplications from the *X* to the autosomes. The causes of the excess X→autosome gene movement are unclear [58], but hypotheses include both mutation bias [58] and selective pressures. Two selection models suggest that X→autosome duplications are compensatory adaptations to either the suboptimal expression levels achievable by *X*-linked testes-expressed genes subjected to MSCI, or to the presence of sexually antagonistic genetic variation [41],[59],[60]. Given the present findings, the general lack of *X* chromosome dosage compensation in the testes provides a more plausible impetus for the evolution of compensatory gene duplications with testes-specific expression than MSCI.

Regardless of its causes, if evolutionary demasculinization has been sufficiently powerful to shape *X* chromosome gene content, then it might cause the *X* chromosome to show lower expression than the autosomes in the male germline as “male-biased genes” in *Drosophila* are largely comprised by those expressed in testes. The challenge, then, is to distinguish the relative contributions of evolutionary demasculinization versus the absence of *X* chromosome dosage compensation to the *X*-autosome difference in expression in *Drosophila* testes. To highlight the difficulty of this problem, it is worth noting that in male-like *SL2* cells in which *X* chromosome dosage compensation has been knocked down, the *X* appears “demasculinized” relative to controls: *msl2*-RNAi cells show a significant deficit of highly expressed genes (and a corresponding excess of lowly expressed genes) on the *X* due to its overall shift towards lower expression (Figure S2).

The two alternatives—evolutionary demasculinization and the lack of *X* chromosome dosage compensation—are not, however, mutually exclusive. Indeed, there is evidence for demasculinization of the *X* in male somatic tissue: fewer than 2% of genes encoding accessory gland proteins reside on the *X* [60]; and a significant, albeit much weaker, signal of a demasculinized *X* is found in microarray analyses of gonadectomized males [3],[61]. In the *Drosophila* testes, however, there is reason to believe that the lack of *X* chromosome dosage compensation is a major determinant of the *X*-autosome difference in expression. In particular, the magnitude of the *X*-autosome difference, whether measured in dissected premeiotic cells or in whole testes, is strikingly similar to that seen for cell lines in which *X* chromosome dosage compensation is experimentally removed. It is unclear why demasculinization should result in so coincidental an *X*-autosome difference in expression. Future analyses of desmasculinization using gene expression data must take into account the lack of *X* chromosome dosage compensation in the *Drosophila* male germline.

Our final caveat is that despite the inability to detect strong, mammal-like MSCI in flies, there is suggestive evidence from cytological analyses. In early primary spermatocytes, in which transcription is active, the heterochromatin-associated H4Ac12 is absent from the three major chromatin clusters, whereas in late spermatocytes H4Ac12 seems to be enriched on the X-Y cluster, suggestive of an increase in heterochromatin on the sex chromosomes [18]. Conversely, H3K4me3, a modification associated with active transcription, appears depleted on the *X* and *Y* late spermatocytes [35]. Given these observations, we suggest that it is formally possible that some form of MSCI exists in flies, that it may even be essential for fertility, but that it simply fails to register in gene expression assays (see also [57]) or at the resolution that the current dissection approaches provide. As we argue below, however, any putative effects of MSCI in meiosis I spermatocytes in *Drosophila* are distinct from those revealed by the expression of the *ocnus* transgene constructs.

### *X* Chromosome and Autosomal Expression of Testes-Specific Transgenes in the *Drosophila* Male Germline

Our stage-specific analysis of *ocnus* transgenes reveals that their striking ∼30-fold *X*-autosome difference in expression is established prior to meiosis I and cannot therefore be attributed to a mammal-like pachytene-specific MSCI. This reduction in *X*-linked transgene expression is neither a consequence of transgene dose nor of meiotic silencing of unpaired chromatin (MSUC) [63], as males heterozygous for autosomal inserts express the transgenes at least as highly as homozygous males (Figure 5A). Furthermore, the *X*-autosome difference cannot be attributed to the absence of germline dosage compensation, for two reasons. First, the *X*-autosome difference is simply too large. Second, single-copy hemizygous *X-*linked inserts are expressed at much lower levels than single-copy heterozygous autosomal ones (Figure 5) [37]. As neither MSCI, MSUC, nor the absence of germline dosage compensation can account for the *X*-autosome difference, we infer that some other, previously undescribed mechanism reduces expression driven by normally autosomal testes-specific promoters from *X*-linked transgenes, a process that begins in premeiotic cells and persists into later stages of spermatogenesis.

The suppression of *X*-linked transgene reporters driven by autosomal testes-specific promoters is not specific to *ocnus* as Hoyle et al. [40] reported similar findings using another autosomal testes-specific promoter, *β2*-*tubulin*. More generally still, the opposite experiment—moving normally *X-*linked testes-specific promoters to autosomal sites—has revealed the opposite effect: when inserted onto autosomes, *X*-linked testes-specific promoters drive *over*-expression in both premeiotic and meiotic cells of *Drosophila* testes (J. Parsch, personal communication). These findings support the notion of a strong, general, *X*-autosome difference in the expression of transgene reporters in the male germline. Anecdotal observations suggest that a similarly dramatic *X*-autosome difference in transgene expression does not occur in the soma [40],[64],[65].

There is a conspicuous discrepancy between the expression of transgene constructs and the expression of endogenous genes in the testes as measured by microarrays or RNA-seq: *ocnus* transgenes show ∼30-fold lower expression from *X-*linked than autosomal inserts, whereas endogenous *X*-linked genes show only ∼1.5-fold lower expression compared to autosomal ones. There are at least two possible explanations for the discrepancy. First, the promoters of spermatogenesis genes encoded on the *X* may have evolved to mitigate the suppressive environment of the *X*. One interpretation of the transgene data, then, is that naïve promoters of autosomal male germline-expressed genes, like *ocnus* and *β2-tubulin*, are not adapted to the *X* and consequently suffer strongly reduced expression when moved to its suppressive environment. Second, the *X*-autosome difference may be specific to expression from transgenes. Such suppression might result from *P*-element transposon sequences that are inserted during transgene integration. If so, it would be, to our knowledge, the first example of germline- and chromosome-specific regulation of expression due to transposon sequences.

### Implications for Speciation in *Drosophila*

Sex chromosomes play a special role in speciation. In *Drosophila*, the sterility of hybrid males is an early and nearly obligate phase in the evolution of complete reproductive isolation between species [66],[67]. The *X* chromosome contributes disproportionately to hybrid male sterility [68],[69], and fine-scale genetic analyses show that the density of genetic factors causing hybrid male sterility is 2.5–4 times higher on the *X* than on the autosomes [70],[71]. One hypothesis for why the *X* is a hotspot for hybrid male sterility is that its regulation in the male germline may be disrupted in hybrids [6],[72],[73]. In the house mouse, for instance, MSCI appears to be disrupted in sterile hybrid males [74],[75]. In *Drosophila,* the absence of dosage compensation in the male germline excludes its disruption as a contributor to hybrid male sterility [69], while disruption of MSCI (if it exists) remains a formal possibility. Gene expression studies of hybrid male sterility in *Drosophila* do not indicate global misregulation of the *X* but, for hybrid males between some species pairs, suggest a slight excess of overexpressed *X*-linked genes [76].

Disruption of *X* chromosome regulation as a basis for hybrid sterility raises the question of what might cause its molecular basis to diverge between species in the first place. The drive hypothesis posits that MSCI evolved as a general mechanism to suppress expression of selfish meiotic drive (segregation distortion) elements on the *X* chromosome [28]–[31]. The *X*-chromosomal transgene suppression we observe here may have evolved for similar reasons. There is increasing evidence that species' genomes harbor cryptic sex chromosome drive elements—drive elements that arose, spread within species, and later came under the control of suppressors [77]. Consistent with the drive model, male mice with genetically compromised MSCI preferentially transmit *X* chromosomes, producing an excess of daughters, as expected if silent distorters on the *X* were released from suppression [32]. The rapid divergence between species might therefore result from antagonistic coevolution between meiotic drivers and the loci controlling these chromosome-wide suppressive mechanisms.

The possibility that recurrent bouts of drive and suppression can cause divergence between species that contributes to hybrid sterility has now been confirmed. Two of the four known hybrid male sterility genes in *Drosophila* are directly involved, either causing sex chromosome drive [78] or suppressing it [79]. A third hybrid male sterility factor, the *X*-linked *Odysseus* (*Ods*) gene [80], behaves like a relict driver: the ODS protein from *Drosophila mauritiana* binds the *D. simulans*—but not the *D. mauritiana*—*Y* chromosome [81]. If *Ods* had a history of drive in *D. mauritiana* by targeting and disrupting the *Y* chromosome, then the *D. mauritiana Y* would be expected to lose sequences targeted by *Ods* while the naïve *D. simulans Y* would not. Finally, the first hybrid sterility gene discovered in mammals, mouse *Prdm9*, disrupts MSCI in hybrid males between two house mouse subspecies [75]. These findings are consistent with a model in which recurrent conflict involving *X* chromosome drive elements, the MSCI machinery in mammals [74], and driver-specific genic suppressors can cause molecular genetic divergence between species that contributes to the rapid evolution of hybrid male sterility.

## Materials and Methods

### Fly Strains

*WOL* and *YLZ* transgene insert lines (described in [37]) were generously provided by John Parsch. All flies were raised on standard cornmeal media at room temperature.

### Sample Preparation for Microarrays

Wild-type individuals of the *OreR* lab strain were used for tissue dissection and RNA extraction. All dissections were done on 1–6-d-old mated males or females. Testis apical tips were dissected in Ringer's solution following [41], except that the surrounding testes sheath was removed. Thoraxes were dissected away from the head and abdomen in Ringer's solution and the legs and wings were removed. All dissected tissue was frozen at −80 until RNA extraction. RNA was extracted using the Clonetech Nucleospin RNA kit following the manufacturer's protocol (including a DNase treatment). Tissue from approximately 40 testis dissections and 100 thoraxes was used per extraction column, and approximately 760 testis apical tips, 100 male thoraxes, and 100 female thoraxes were used for each microarray hybridization. RNA extractions were pooled into four independent samples, and 1 µg of total RNA was used as a template for cRNA synthesis with Ambion's Amino Allyl MessageAmp aRNA amplification protocol. Cy3 labeled cRNA was hybridized to Agilent *Drosophila* gene expression microarrays and scanned with an Agilent G2505B scanner. cRNA synthesis and array hybridization were done at the Cornell Microarray Core Facility. Array data are available at the NCBI GEO under accession # GSE30850.

### Microarray Analysis

Background subtracted probe intensities calculated by Agilent software were used as raw signal intensity values. Signal intensity was averaged across replicate spots for probes represented more than once on the array. Probe-level log2 signal intensities were used to estimate expression levels for each probe in each of the three tissues (male thorax, female thorax, male germline). All analyses were done with the limma package [82] in R [83].

Previously published gene expression data from testes dissections were obtained from Supplementary Table 1 of Vibranovski et al. [41]. Statistically significant gene expression differences between spermatogenic stages were determined by probe set *t* tests, corrected for multiple tests by controlling the FDR [52]. The distributions of signal intensities on both these arrays and the Agilent arrays are distinctly bimodal (Figure S1). Vibranovski et al. [41] did not exclude genes that are lowly expressed (and thus unreliably measured on the microarray) or not significantly expressed above background, and thus the lower mode likely includes noise that may obscure real differences between expression of *X*-linked and autosomal genes [43],[56]. Therefore, all probe sets with log2 expression levels <6 in all three dissections from [41] were removed when calculating *X*-autosome ratios of expression. Similarly, all probes on the Agilent arrays with log2 expression levels<7 in all three tissues (male thorax, female thorax, male germline) were removed when calculating *X*-autosome ratios of expression (see Figure S1).

### Somatic Contamination qRT-PCR

Premeiotic germline cells in the apical tip of the testis were dissected in Ringer's solution either including the somatic cells of the testis sheath (following [41] exactly), or they were removed from the sheath in a manner similar to the meiotic (proximal) dissections of Vibranovski et al. [41]. Meiotic cells were dissected following [41]. Approximately 50 dissections from each cell type (apical cells with and without testis sheath and proximal cells without testis sheath) were used for RNA extraction with the Clonetech Nucleospin RNA kit. 5 µL of eluted RNA was used as a template for cDNA synthesis with Superscript III (Invitrogen) and primed with oligo-dT. 1 µL of cDNA was used in a 20 uL qRT-PCR reaction with ABI Taqman probes. Two replicate qRT-PCR reactions were run on a 96-well plate, and each plate was run in duplicate. Ct values were averaged across replicate wells within a plate for each probe, and the mean Ct value for the three control genes within each dissection on each plate was calculated to control for the amount of RNA in each dissection. Normalized Ct values for target genes were obtained by subtracting mean control gene Ct values.

### Whole Fly *ocn* Transgene qRT-PCR

Approximately 10 young adult male and female flies of each genotype were flash-frozen in liquid nitrogen and RNA was extracted using a standard TRIzol/chloroform protocol, followed by an EtOH precipitation. At least 3 ug of RNA was used as a template for cDNA synthesis. 1 µL of cDNA was used in a 20 µL qRT-PCR reaction with ABI Taqman probes complementary to the *ocn*::*lacZ* transgene or *RpL32* as a control (these are the same probes used by Hense et al. [37]). Three replicate reactions were run on a single plate and Ct values were averaged across replicate wells for the transgene and control probes. The *Anova* function in the *car* package in R was used for a factorial ANOVA with Type II sums of squares and the following model: Normalized Ct ∼ Sex * Location (*X* versus A) * Transgene (*WOL* versus *YLZ*).

### Spermatogenic Stage-Specific *ocn* Transgene qRT-PCR

RNA was extracted from approximately 50 dissected testes of apical (premeiotic) cells with the sheath removed or proximal (meiotic) cells using the Clonetech Nucleospin RNA kit. Five replicate dissections for each spermatogenic stage were done for each genotype. Five µL of eluted RNA were used for cDNA synthesis and 1 µL of cDNA was used for a 20 µL qRT-PCR reaction with ABI Taqman probes. Two replicate qRT-PCR reactions were run for each cDNA sample on a single plate, and each plate was run in duplicate. Ct values for target and control genes were averaged across wells and plates to produce a single value used in a factorial ANOVA with Type II sums of squares and the following model: Normalized Ct ∼ Tissue (premeiotic versus meiotic) * Location (*X* versus A) * Transgene (*WOL* versus *YLZ*).

### RNA-Seq Analysis of Wild-Type and *bam* Mutant Testes and Ovaries

RNA-seq data from [48] were obtained from the Short Read Archive (NCBI). We aligned sequences to the *D. melanogaster* genome (version 5.22) using TopHat [84]; splice junctions were obtained from a GTF file of the *D. melanogaster* transcriptome downloaded from Ensembl (http://useast.ensembl.org/info/data/ftp/index.html). Transcripts were assembled and their abundances estimated with Cufflinks [85]. Summary statistics of the mapping and assembly are provided in Table S7. For the purpose of calculating *X* and autosome expression, RPKM values were summed across all transcripts matching the same gene, and only genes with RPKM values >1 were included in the analysis.

All data analyses were done in R [83].

## Supporting Information

Figure S1 Gene expression in male and female thorax is highly correlated.
(PDF)

Figure S2 Distributions of *X* chromosome and autosomal gene expression levels for six microarray data sets analyzed in this study. Each distribution was divided into 10 quantiles including both *X-*linked and autosomal probes, and statistics associated with all probes expressed at or higher than each quantile are reported above the histograms. For example, the column labeled “>q90” lists the number of *X-*linked and autosomal probes in the top 10% of all probes in that microarray experiment; the proportion of these probes that are *X-*linked; the *p-*value from a Fisher's exact test contrasting the number of *X*-linked and autosomal probes in the top 10% with the values from the bottom 90% of probes; the difference between the median expression value of autosomal and *X-*linked probes in the top 10%; and the *p*-value from a Mann-Whitney test of these median values. Dark black lines indicate values at which distributions were truncated for calculating overall differences in expression between *X* and autosomal probes (see text). (A), (C), and (D) are from Agilent microarrays reported here; (B) is from the mitotic dissections and Affymetrix microarray study of Vibranovski et al. 2009 [41]; (E) and (F) are from the MSL2 RNAi Affymetrix microarray study of Hamada et al. 2005 [43].
(PDF)

Figure S3 Volcano plots of microarray analysis of dissected male germline tissue from Vibranovski et al. 2009 [41]. In the top two panels, the -log_10_ *p*-value from probe-level *t*-tests are plotted against the magnitude of differential expression between premeiotic and meiotic cells (A) and meiotic and postmeiotic cells (B), averaged across three replicate arrays. In (C) and (D), log-transformed Bayesian posterior probabilities reported in Vibranovski et al. 2009 [41] (their Supplementary Table 1) are plotted against the same changes in expression. 10^−4^ was added to the Bayesian probability values to allow plotting probes for which the Bayesian probability was 0. The *t*-tests are more sensitive to probe-specific variation between replicate arrays than the previously reported Bayesian analysis.
(PDF)

Table S1 Number of genes with significant differences in expression between stages of spermatogenesis (FDR = 0.01).
(PDF)

Table S2 Median log2 magnitude of changes in expression between stages of spermatogenesis (FDR = 0.01).
(PDF)

Table S3 Number of genes with significant differences in expression between stages of spermatogenesis (FDR = 0.005).
(PDF)

Table S4 Median log2 magnitude of changes in expression between stages of spermatogenesis (FDR = 0.005).
(PDF)

Table S5 Number of genes with significant differences in expression between stages of spermatogenesis (2-fold cutoff).
(PDF)

Table S6 Median log2 magnitude of changes in expression between stages of spermatogenesis (2-fold cutoff).
(PDF)

Table S7 RNAseq statistics.
(PDF)

## Abbreviations

DCC: dosage compensation complex
FDR: false discovery rate
MSCI: meiotic sex chromosome inactivation
MSUC: meiotic silencing of unpaired chromatin
RNAi: RNA interference
