# Supplementary figures and images for: Sex Chromosome-Specific Regulation in the Drosophila Male Germline But Little Evidence for Chromosomal Dosage Compensation or Meiotic Inactivation
Source: PLoS Biol. 2011 Aug 16;9(8):e1001126. doi: 10.1371/journal.pbio.1001126 (PMC3156688; doi:10.1371/journal.pbio.1001126)

**all probes**

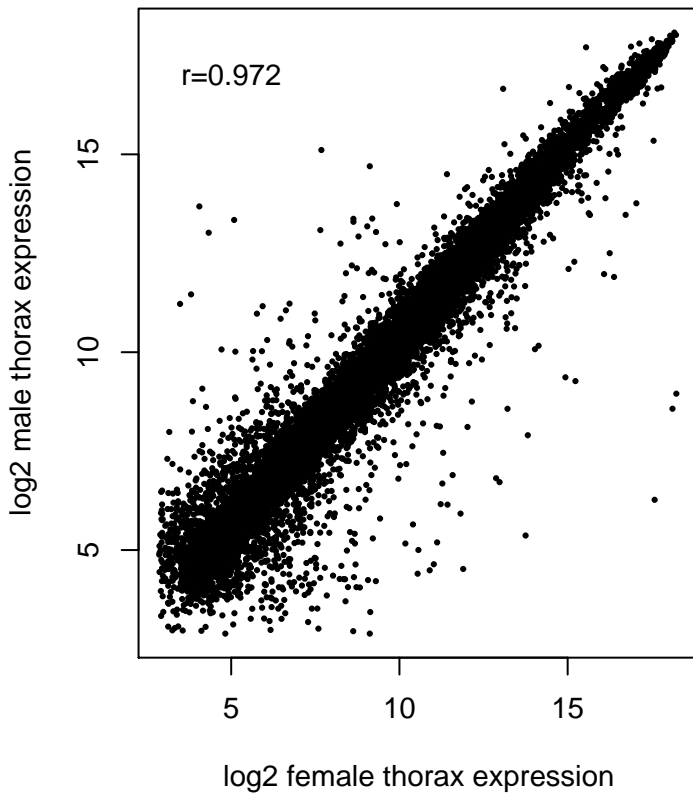

**autosomal probes**

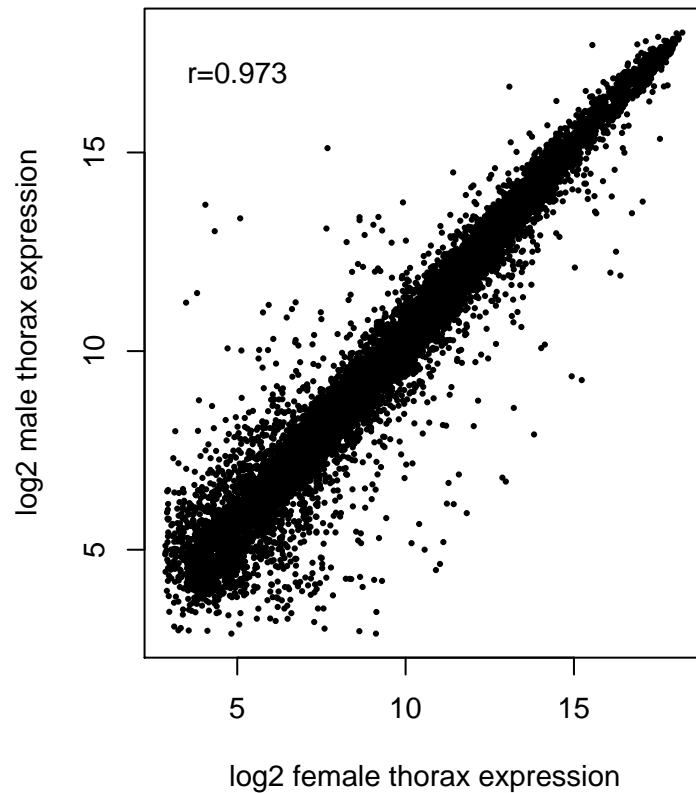

**X-linked probes**

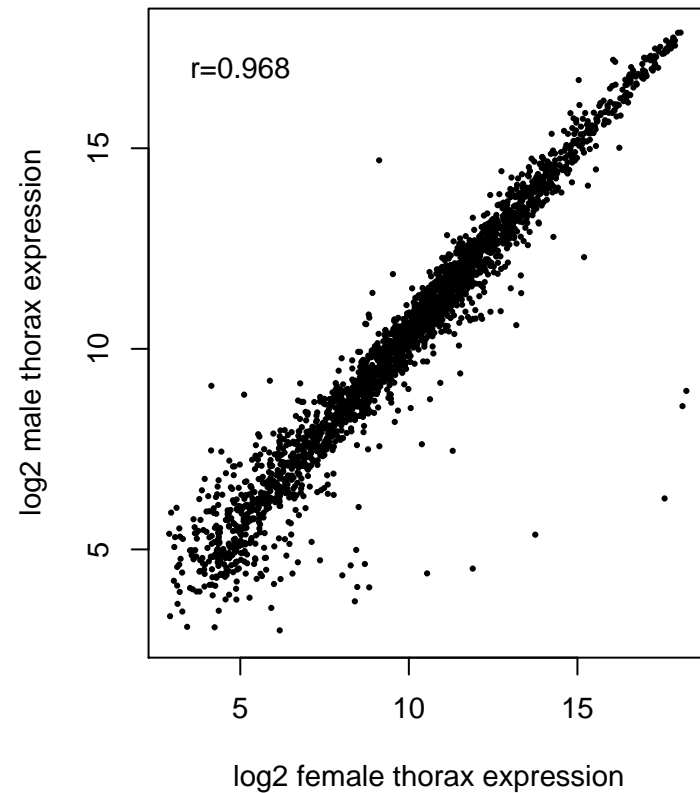

Supplement: Figure S1 — Gene expression in male and female thorax is highly correlated. (PDF) [file pbio.1001126.s001.pdf]

Supplementary Figure 3

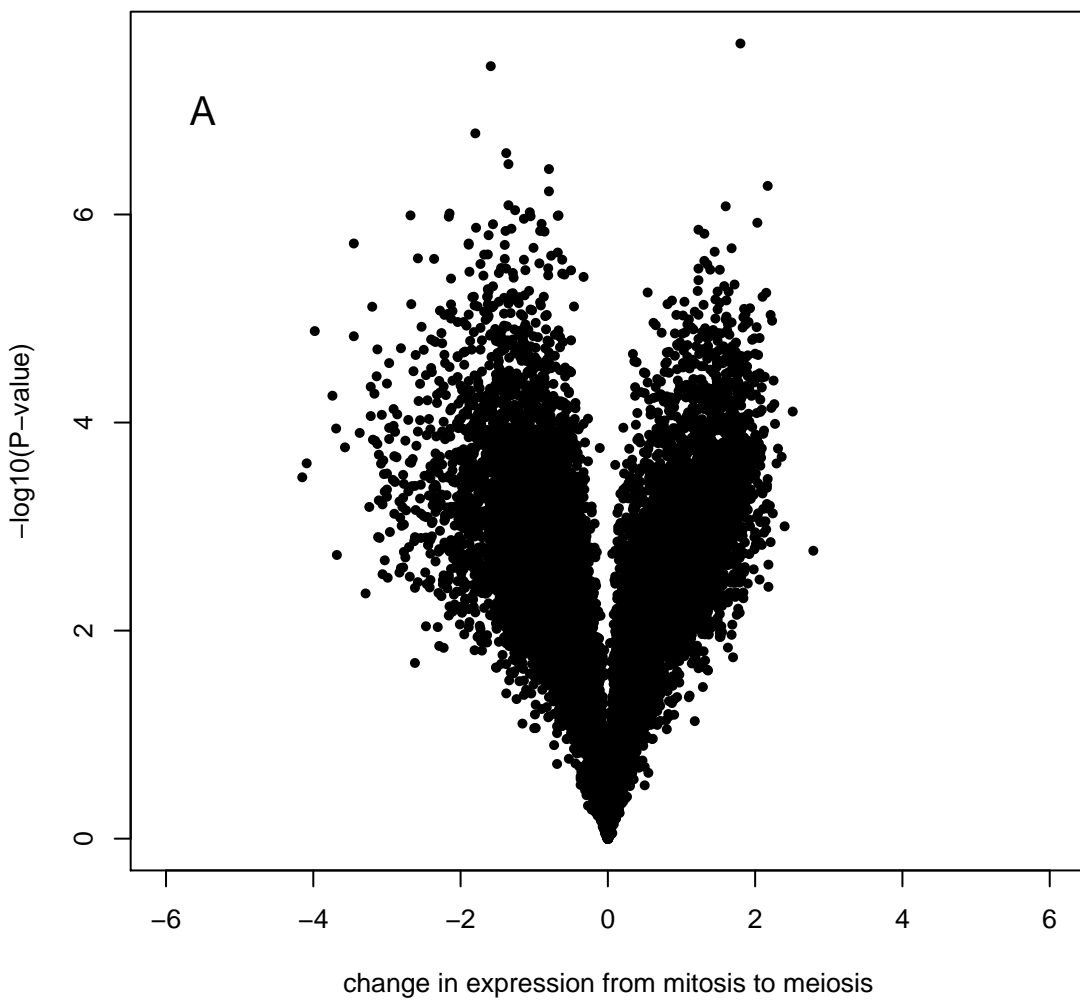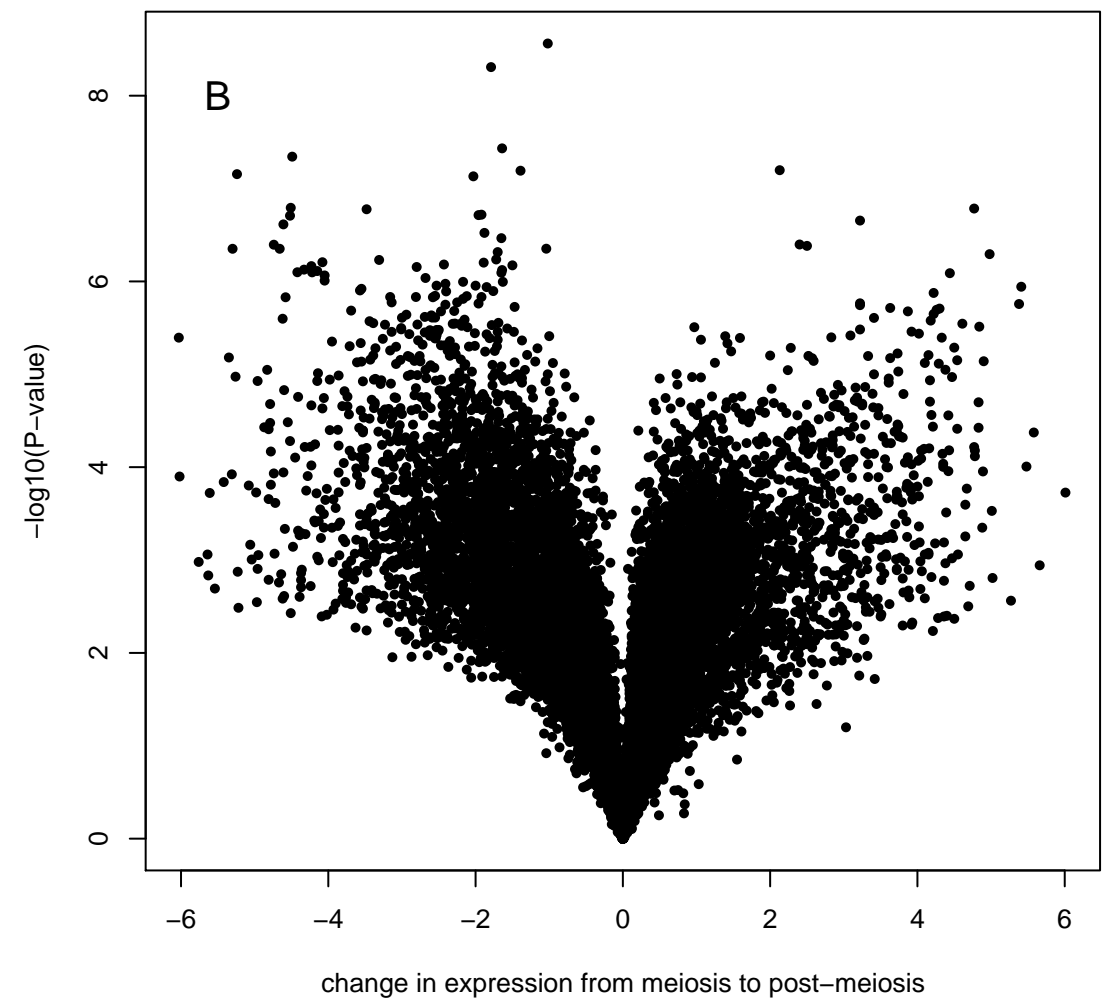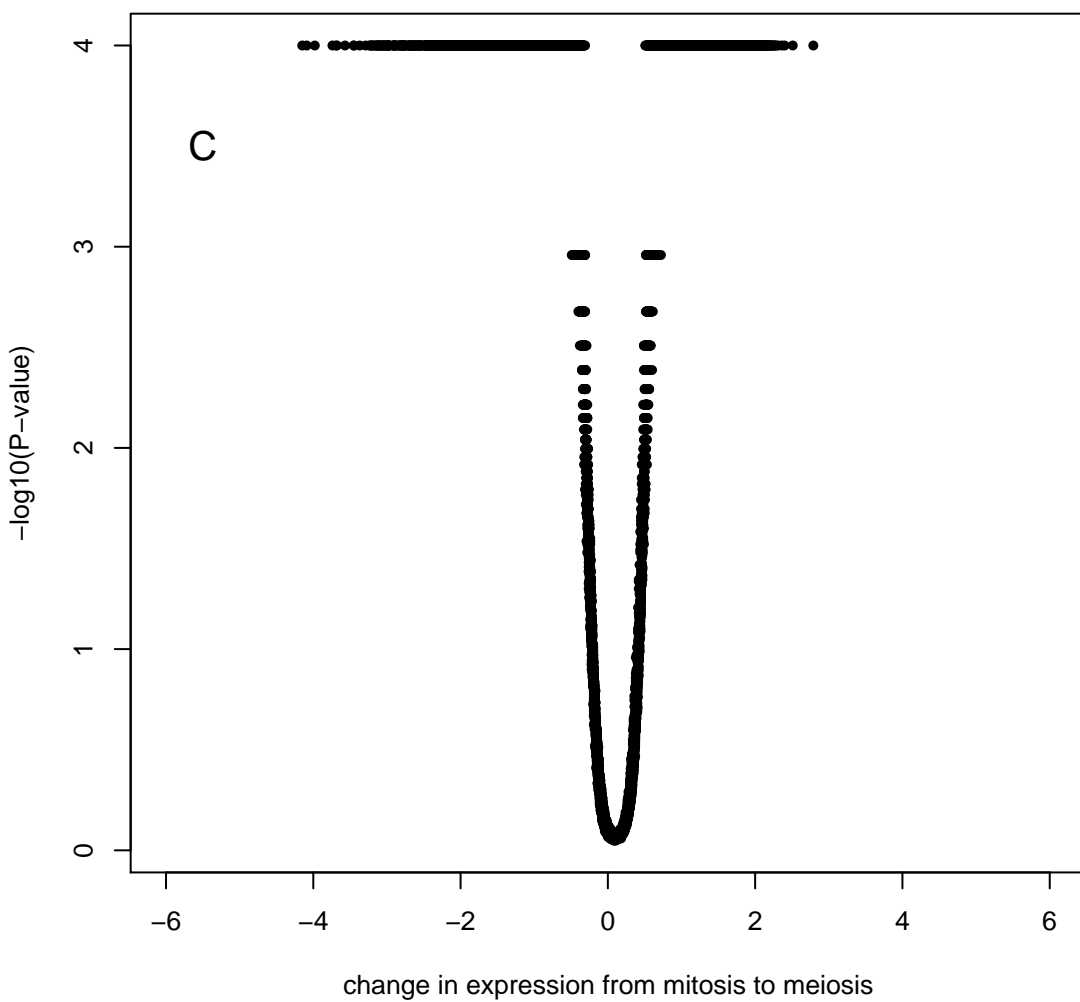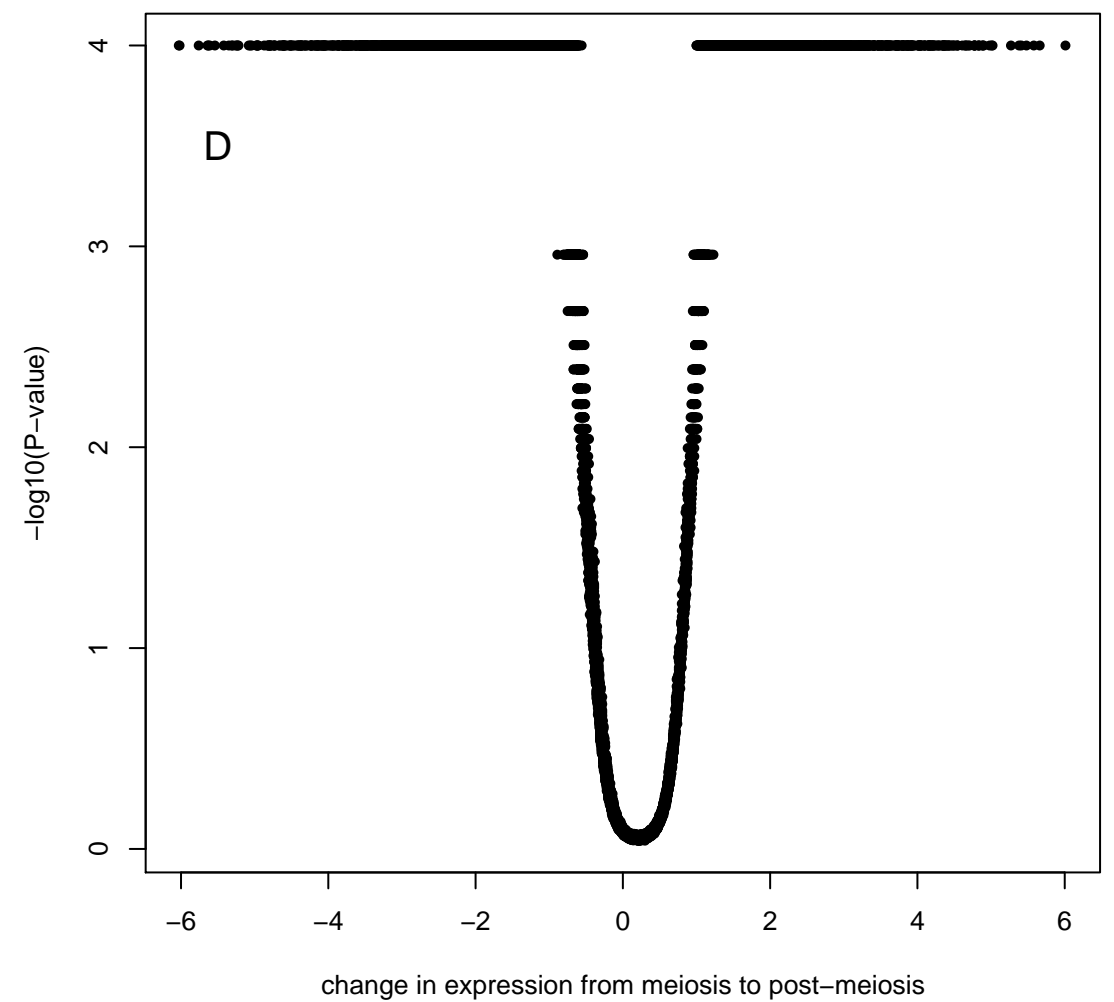

Supplement: Figure S3 — Volcano plots of microarray analysis of dissected male germline tissue from Vibranovski et al. 2009 [41]. In the top two panels, the -log10 p-value from probe-level t-tests are plotted against the magnitude of differential expression between premeiotic and meiotic cells (A) and meiotic and postmeiotic cells (B), averaged across three replicate arrays. In (C) and (D), log-transformed Bayesian posterior probabilities reported in Vibranovski et al. 2009 [41] (their Supplementary Table 1) are plotted against the same changes in expression. 10−4 was added to the Bayesian probability values to allow plotting probes for which the Bayesian probability was 0. The t-tests are more sensitive to probe-specific variation between replicate arrays than the previously reported Bayesian analysis. (PDF) [file pbio.1001126.s003.pdf]
