# Supplementary material for: Sex Chromosome-Specific Regulation in the Drosophila Male Germline But Little Evidence for Chromosomal Dosage Compensation or Meiotic Inactivation
Source: PLoS Biol. 2011 Aug 16;9(8):e1001126. doi: 10.1371/journal.pbio.1001126 (PMC3156688; doi:10.1371/journal.pbio.1001126)

Supplementary Figure 2A: mitotic germline (Agilent)

all probes	>q10	>q20	>q30	>q40	>q50	>q60	>q70	>q80	>q90	
2995	2661	2391	2076	1741	1373	1066	759	486	235	# X probes
15362	13861	12295	10774	9274	7806	6277	4749	3186	1601	# A probes
0.16	0.16	0.16	0.16	0.16	0.15	0.15	0.14	0.13	0.13	% X−linked
–	1.6	0.1	0.4	1.6	6.2	7.2	9.1	8	5	−log10(P−value)
0.48	0.58	0.62	0.58	0.5	0.36	0.26	0.18	0.17	0.05	median A−X
7.7	6.4	11.9	14.5	14.9	8.5	7.6	4.7	4.2	1	−log10(P−value)

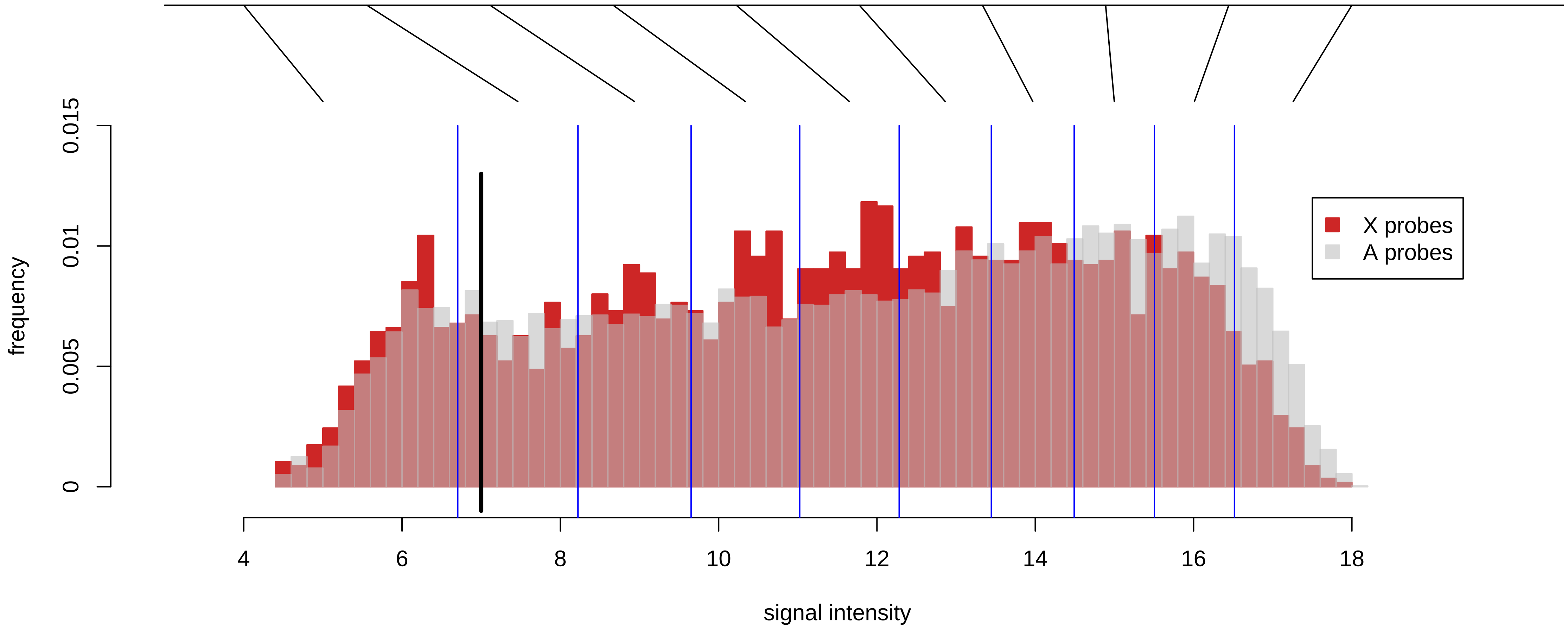

Supplementary Figure 2B: mitotic germline (Affymetrix)

all probes	>q10	>q20	>q30	>q40	>q50	>q60	>q70	>q80	>q90	
2982	2684	2406	2096	1796	1467	1107	727	422	190	# X probes
15099	13569	12073	10553	9051	7568	6130	4691	3200	1618	# A probes
0.16	0.17	0.17	0.17	0.17	0.16	0.15	0.13	0.12	0.11	% X−linked
–	0.1	0.4	0.2	0.1	0.4	3.4	12.8	>15	13.5	−log10(P−value)
0.06	0.14	0.37	0.55	0.67	0.6	0.52	0.34	0.14	0.15	median A−X
3.8	5.2	8.6	12.1	>15	>15	>15	9.9	3.1	1.9	−log10(P−value)

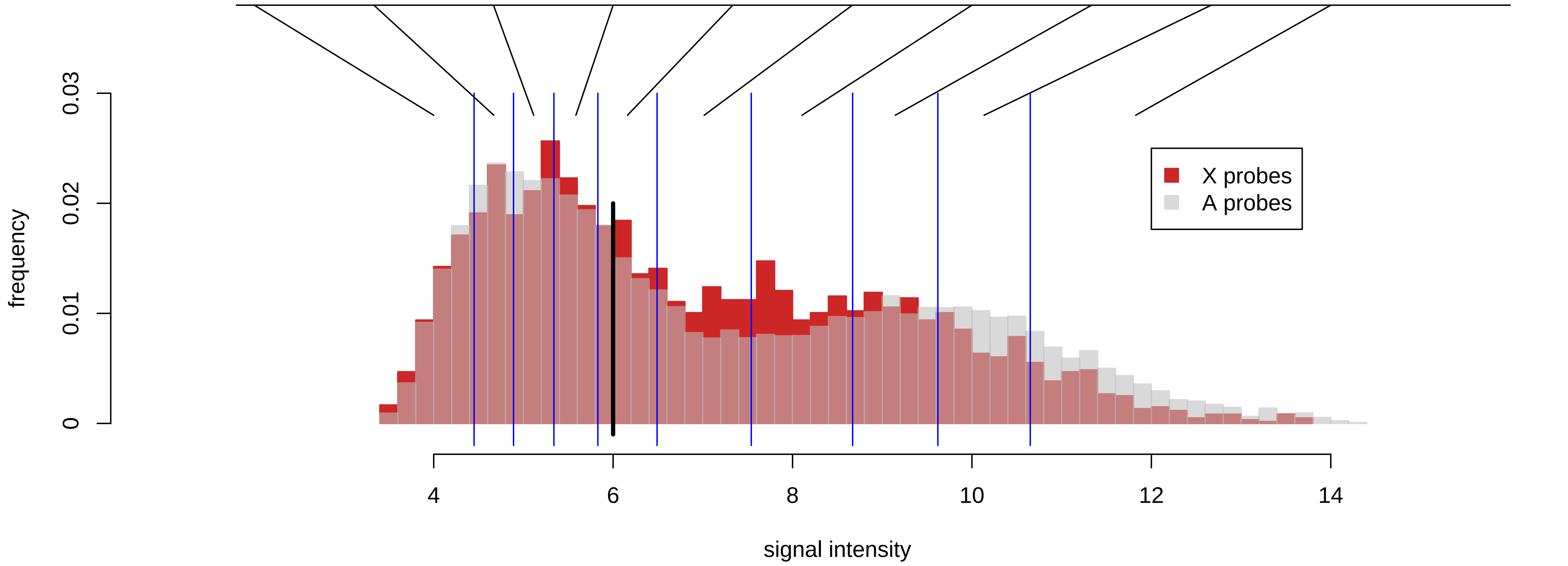

Supplementary Figure 2C: male thorax

all probes	>q10	>q20	>q30	>q40	>q50	>q60	>q70	>q80	>q90	
2995	2698	2382	2082	1786	1469	1124	808	529	265	# X probes
15362	13824	12304	10768	9229	7710	6219	4700	3143	1571	# A probes
0.16	0.16	0.16	0.16	0.16	0.16	0.15	0.15	0.14	0.14	% X−linked
–	0	0.3	0.3	0.2	0.6	2.6	4.1	3.4	1.7	−log10(P−value)
0.02	0.12	0.17	0.19	0.27	0.28	0.15	0.04	−0.05	−0.1	median A−X
1.1	1.6	1.5	2	3.5	4	1.4	0.1	0.4	0.5	−log10(P−value)

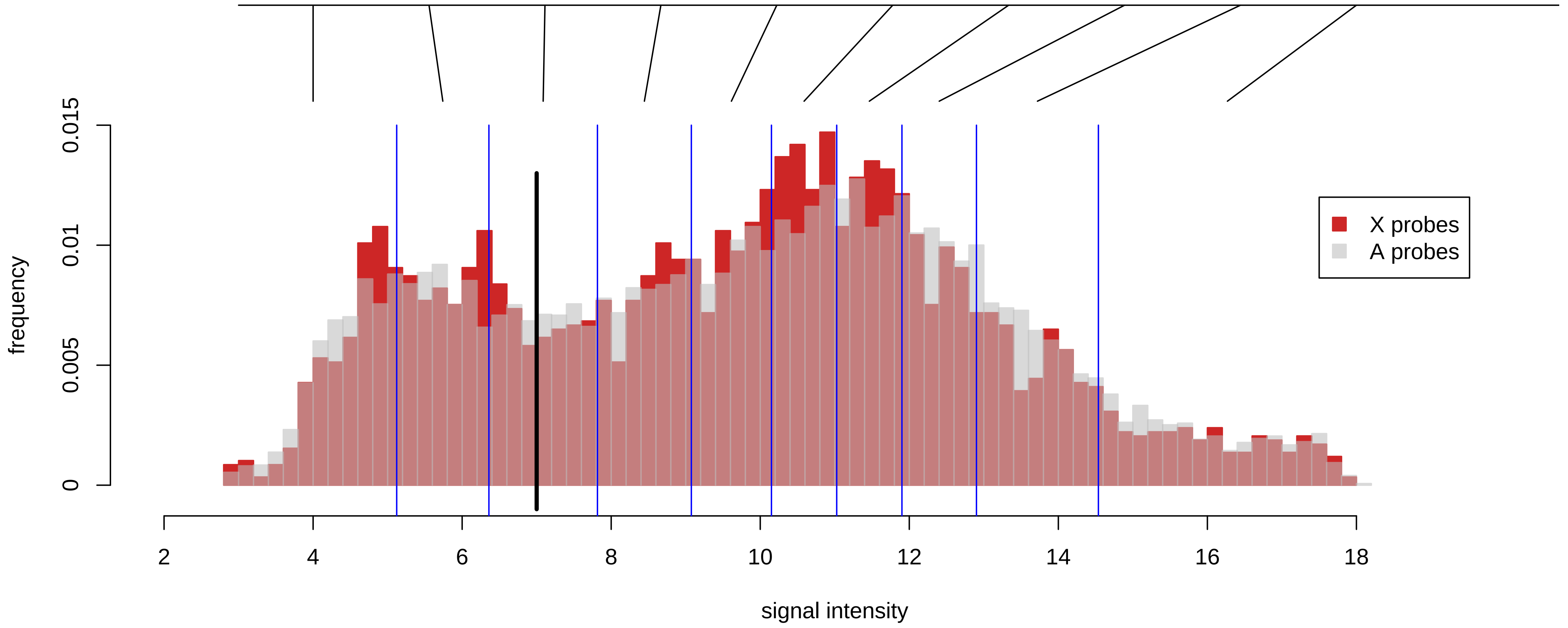

Supplementary Figure 2D: female thorax

all probes	>q10	>q20	>q30	>q40	>q50	>q60	>q70	>q80	>q90	
2995	2693	2357	2085	1767	1434	1121	796	525	260	# X probes
15362	13829	12329	10765	9248	7745	6222	4712	3147	1576	# A probes
0.16	0.16	0.16	0.16	0.16	0.16	0.15	0.14	0.14	0.14	% X−linked
–	0.1	1.3	0.2	0.7	2	2.8	5.2	3.7	2.1	−log10(P−value)
0.13	0.17	0.14	0.2	0.25	0.2	0.16	−0.06	−0.05	0.02	median A−X
1.4	1.8	0.8	2.7	3	1.9	1.3	0.4	0.8	0.3	−log10(P−value)

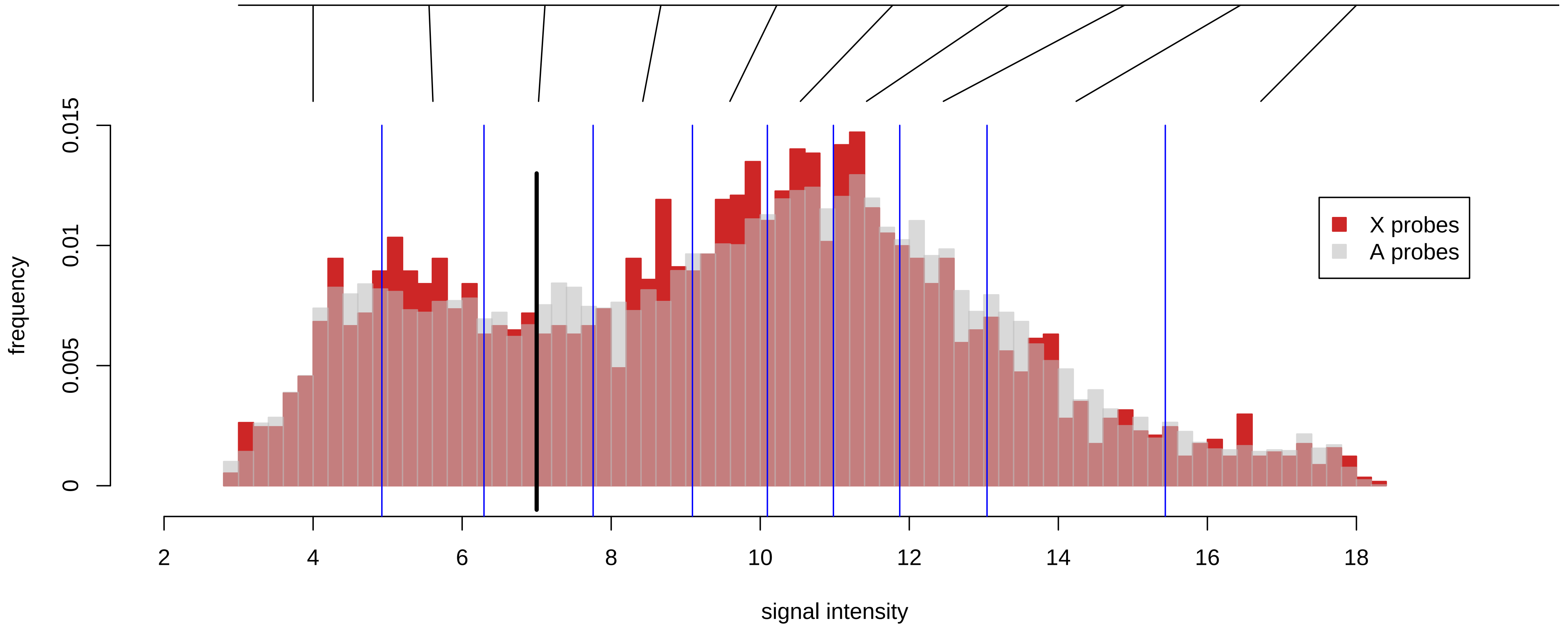

Supplementary Figure 2E: MSL2 RNAi (Hamada)

all probes	>q10	>q20	>q30	>q40	>q50	>q60	>q70	>q80	>q90	
1297	1139	973	789	645	507	384	259	174	80	# X probes
6309	5707	5112	4536	3919	3296	2659	2023	1348	681	# A probes
0.17	0.17	0.16	0.15	0.14	0.13	0.13	0.11	0.11	0.11	% X−linked
–	2.3	5.8	14	>15	>15	>15	>15	10.8	6.8	−log10(P−value)
0.59	0.55	0.44	0.3	0.31	0.29	0.14	0.04	0.13	−0.15	median A−X
>15	>15	>15	9.2	6.9	4.1	2.9	0.3	0.6	0.1	−log10(P−value)

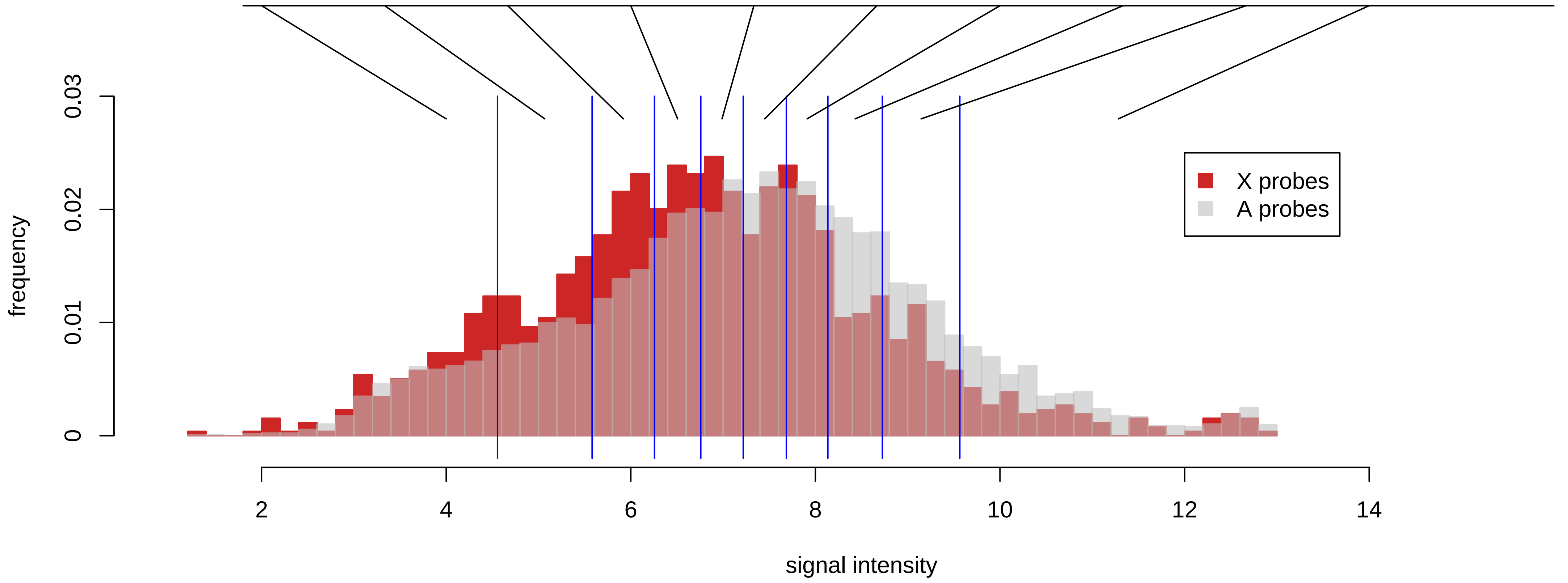

Supplementary Figure 2F: control cells (Hamada)

all probes	>q10	>q20	>q30	>q40	>q50	>q60	>q70	>q80	>q90	
1297	1167	1034	885	743	599	478	351	216	106	# X probes
6309	5679	5051	4440	3821	3204	2565	1931	1306	655	# A probes
0.17	0.17	0.17	0.17	0.16	0.16	0.16	0.15	0.14	0.14	% X−linked
–	0	0.1	0.9	1.5	2.6	1.9	1.9	3.1	1.8	−log10(P−value)
0.2	0.2	0.16	0.1	0.09	0.04	0.11	0.04	0.04	0.03	median A−X
2	2.7	3.3	2.2	1.6	0.6	0.8	0.7	0.2	0.2	−log10(P−value)

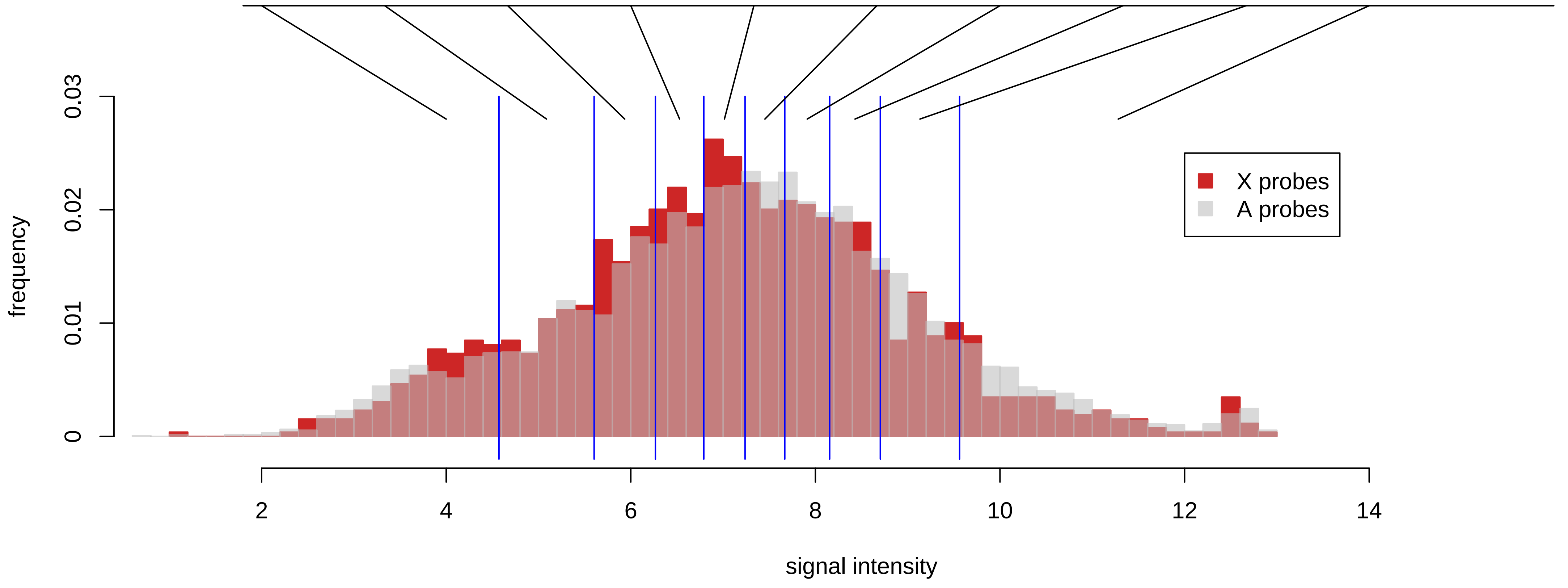

Supplement: Figure S2 — Distributions of X chromosome and autosomal gene expression levels for six microarray data sets analyzed in this study. Each distribution was divided into 10 quantiles including both X-linked and autosomal probes, and statistics associated with all probes expressed at or higher than each quantile are reported above the histograms. For example, the column labeled “>q90” lists the number of X-linked and autosomal probes in the top 10% of all probes in that microarray experiment; the proportion of these probes that are X-linked; the p-value from a Fisher's exact test contrasting the number of X-linked and autosomal probes in the top 10% with the values from the bottom 90% of probes; the difference between the median expression value of autosomal and X-linked probes in the top 10%; and the p-value from a Mann-Whitney test of these median values. Dark black lines indicate values at which distributions were truncated for calculating overall differences in expression between X and autosomal probes (see text). (A), (C), and (D) are from Agilent microarrays reported here; (B) is from the mitotic dissections and Affymetrix microarray study of Vibranovski et al. 2009 [41]; (E) and (F) are from the MSL2 RNAi Affymetrix microarray study of Hamada et al. 2005 [43]. (PDF) [file pbio.1001126.s002.pdf]
