# Supplementary material for: Sex Chromosome-Specific Regulation in the Drosophila Male Germline But Little Evidence for Chromosomal Dosage Compensation or Meiotic Inactivation
Source: PLoS Biol. 2011 Aug 16;9(8):e1001126. doi: 10.1371/journal.pbio.1001126 (PMC3156688; doi:10.1371/journal.pbio.1001126)
Supplement: Table S1 — Number of genes with significant differences in expression between stages of spermatogenesis (FDR = 0.01). (PDF) [file pbio.1001126.s004.pdf]

Supplementary Table 1. Number of genes with significant differences in expression between stages of spermatogenesis (FDR = 0.01)

FDR = 0.01		Early changes (premeiosis:meiosis)		Late changes (meiosis:postmeiosis)		Net change (premeiosis:postmeiosis)	
chromosomal arm	# expressed	down	up	down	up	down	up
2L	2204	516 (23.4%)	478 (21.7%)	574 (26.0%)	370 (16.8%)	731 (33.2%)	561 (25.5%)
2R	2356	625 (26.5%)	467 (19.8%)	549 (23.3%)	410 (17.4%)	754 (32.0%)	595 (32.0%)
3L	2335	586 (25.1%)	461 (19.7%)	544 (23.3%)	374 (16.0%)	740 (31.7%)	593 (25.4%)
3R	3009	744 (24.7%)	534 (17.8%)	694 (23.1%)	475 (15.8%)	959 (31.9%)	720 (23.9%)
4	58	29 (50.0%)	6 (10.3%)	14 (24.1%)	12 (20.7%)	24 (41.4%)	6 (10.3%)
X	1943	487 (25.1%)	**291 (15.0%)**	**361 (18.6%)**	338 (17.4%)	**555 (28.6%)**	441 (22.7%)
A*	9904	2471 (25.0%)	**1940 (19.6%)**	**2361 (23.8%)**	1629 (16.4%)	**3184 (32.1%)**	2469 (24.9%)
X vs A (*FET P*-value)		0.909	**1.37E-06**	**3.26E-07**	0.302	**0.002**	0.038

*autosomal totals exclude genes on the 4th chromosome
