# Supplementary material for: Sex Chromosome-Specific Regulation in the Drosophila Male Germline But Little Evidence for Chromosomal Dosage Compensation or Meiotic Inactivation
Source: PLoS Biol. 2011 Aug 16;9(8):e1001126. doi: 10.1371/journal.pbio.1001126 (PMC3156688; doi:10.1371/journal.pbio.1001126)
Supplement: Table S2 — Median log2 magnitude of changes in expression between stages of spermatogenesis (FDR = 0.01). (PDF) [file pbio.1001126.s005.pdf]

Supplementary Table 2. Median log2 magnitude of changes in expression between stages of spermatogenesis (FDR = 0.01)

FDR = 0.01	Early changes (premeiosis:meiosis)			Late changes (meiosis:postmeiosis)			Net change (premeiosis:postmeiosis)		
chromosomal arm	down	up	down - up	down	up	down - up	down	up	down - up
2L	-1.07	1.21	0.14	-1.68	1.27	-0.42	-1.91	1.52	-0.39
2R	-1.04	1.24	0.20	-1.65	1.33	-0.32	-1.97	1.49	-0.48
3L	-1.01	1.21	0.20	-1.71	1.21	-0.50	-1.92	1.29	-0.63
3R	-1.04	1.18	0.14	-1.68	1.19	-0.49	-1.92	1.42	-0.51
4	-1.08	1.11	0.02	-1.16	2.16	1.00	-1.63	1.72	0.09
X	-1.06	**1.02**	-0.04	**-1.50**	1.28	-0.23	**-1.73**	**1.31**	-0.42
A*	-1.04	**1.21**	0.17	**-1.68**	1.24	-0.44	**-1.93**	**1.43**	-0.50
X vs A (*MW P*-value)	0.376	**5.34E-09**		**5.93E-03**	0.247		**1.64E-04**	**5.49E-03**	

*autosomal totals exclude genes on the 4th chromosome
