# Supplementary material for: Sex Chromosome-Specific Regulation in the Drosophila Male Germline But Little Evidence for Chromosomal Dosage Compensation or Meiotic Inactivation
Source: PLoS Biol. 2011 Aug 16;9(8):e1001126. doi: 10.1371/journal.pbio.1001126 (PMC3156688; doi:10.1371/journal.pbio.1001126)
Supplement: Table S3 — Number of genes with significant differences in expression between stages of spermatogenesis (FDR = 0.005). (PDF) [file pbio.1001126.s006.pdf]

Supplementary Table 3. Number of genes with significant differences in expression between stages of spermatogenesis (FDR = 0.005)

FDR = 0.005		Early changes (premeiosis:meiosis)		Late changes (meiosis:postmeiosis)		Net change (premeiosis:postmeiosis)	
chromosomal arm	# expressed	down	up	down	up	down	up
2L	2204	358 (16.2%)	332 (15.1%)	404 (18.3%)	229 (10.4%)	598 (27.1%)	420 (19.1%)
2R	2356	422 (17.9%)	325 (13.8%)	380 (16.1%)	278 (11.8%)	610 (15.9%)	448 (19.0%)
3L	2335	392 (16.8%)	328 (14.0%)	351 (15.0%)	233 (10.0%)	589 (25.2%)	421 (18.0%)
3R	3009	532 (17.7%)	360 (12.0%)	480 (16.0%)	301 (10.0%)	757 (25.2%)	512 (17.0%)
4	58	22 (37.9%)	5 (8.6%)	7 (12.1%)	8 (13.8%)	21 (36.2%)	5 (8.6%)
X	1943	324 (16.7%)	**195 (10.0%)**	**239 (12.3%)**	215 (11.1%)	**433 (22.3%)**	319 (16.4%)
A*	9904	1704 (17.2%)	**1345 (13.6%)**	**1615 (16.3%)**	1041 (10.5%)	**2554 (25.8%)**	1801 (18.2%)
X vs A (*FET P*-value)		0.598	**1.54E-05**	**6.37E-06**	0.468	**0.001**	0.065

*autosomal totals exclude genes on the 4th chromosome
