# Supplementary material for: Sex Chromosome-Specific Regulation in the Drosophila Male Germline But Little Evidence for Chromosomal Dosage Compensation or Meiotic Inactivation
Source: PLoS Biol. 2011 Aug 16;9(8):e1001126. doi: 10.1371/journal.pbio.1001126 (PMC3156688; doi:10.1371/journal.pbio.1001126)
Supplement: Table S4 — Median log2 magnitude of changes in expression between stages of spermatogenesis (FDR = 0.005). (PDF) [file pbio.1001126.s007.pdf]

Supplementary Table 4. Median log2 magnitude of changes in expression between stages of spermatogenesis (FDR = 0.005)

FDR = 0.005	Early changes (premeiosis:meiosis)			Late changes (meiosis:postmeiosis)			Net change (premeiosis:postmeiosis)		
chromosomal arm	down	up	down - up	down	up	down - up	down	up	down - up
2L	-1.16	1.33	0.18	-1.79	1.52	-0.27	-2.01	1.72	-0.29
2R	-1.17	1.36	0.19	-1.77	1.43	-0.34	-2.10	1.65	-0.45
3L	-1.10	1.35	0.25	-1.93	1.37	-0.56	-2.02	1.55	-0.47
3R	-1.10	1.28	0.18	-1.87	1.41	-0.46	-2.04	1.59	-0.45
4	-1.19	1.13	-0.06	-1.08	2.35	1.27	-1.53	1.74	0.21
X	-1.17	**1.08**	-0.09	**-1.72**	1.54	-0.18	**-1.84**	**1.48**	-0.36
A*	-1.12	**1.33**	0.21	**-1.85**	1.43	-0.42	**-2.04**	**1.61**	-0.43
X vs A (*MW P*-value)	0.126	**3.82E-09**		**1.84E-01**	0.099		**3.21E-04**	**7.15E-03**	

*autosomal totals exclude genes on the 4th chromosome
