# Supplementary material for: Sex Chromosome-Specific Regulation in the Drosophila Male Germline But Little Evidence for Chromosomal Dosage Compensation or Meiotic Inactivation
Source: PLoS Biol. 2011 Aug 16;9(8):e1001126. doi: 10.1371/journal.pbio.1001126 (PMC3156688; doi:10.1371/journal.pbio.1001126)
Supplement: Table S5 — Number of genes with significant differences in expression between stages of spermatogenesis (2-fold cutoff). (PDF) [file pbio.1001126.s008.pdf]

Supplementary Table 5. Number of genes with significant differences in expression between stages of spermatogenesis (2-fold cutoff)

2-fold cutoff		Early changes (premeiosis:meiosis)				Late changes (meiosis:postmeiosis)				Net change (premeiosis:postmeiosis)			
chromosomal arm	# expressed	down		up		down		up		down		up	
2L	2204	318	(14.4%)	353	(16.0%)	545	(24.7%)	305	(13.8%)	707	(32.1%)	471	(21.4%)
2R	2356	380	(16.1%)	351	(14.9%)	553	(23.5%)	361	(15.3%)	712	(30.2%)	507	(21.5%)
3L	2335	329	(14.1%)	337	(14.4%)	535	(22.9%)	302	(12.9%)	723	(31.0%)	434	(18.6%)
3R	3009	441	(14.7%)	371	(12.3%)	714	(23.7%)	406	(13.5%)	943	(31.3%)	590	(19.6%)
4	58	16	(27.6%)	4	(6.9%)	11	(19.0%)	11	(19.0%)	24	(41.4%)	4	(6.9%)
X	1943	318	(16.4%)	**173**	**(8.9%)**	**350**	**(18.0%)**	312	(16.1%)	**540**	**(27.8%)**	**328**	**(16.9%)**
A*	9904	1468	(14.8%)	**1412**	**(14.3%)**	**2347**	**(23.7%)**	1374	(13.9%)	**3085**	**(31.1%)**	**2002**	**(20.2%)**
X vs A (*FET P*-value)		0.083		**4.63E-11**		**2.57E-08**		0.013		**0.003**		**6.65E-04**	

*autosomal totals exclude genes on the 4th chromosome
