# Supplementary material for: Sex Chromosome-Specific Regulation in the Drosophila Male Germline But Little Evidence for Chromosomal Dosage Compensation or Meiotic Inactivation
Source: PLoS Biol. 2011 Aug 16;9(8):e1001126. doi: 10.1371/journal.pbio.1001126 (PMC3156688; doi:10.1371/journal.pbio.1001126)
Supplement: Table S6 — Median log2 magnitude of changes in expression between stages of spermatogenesis (2-fold cutoff). (PDF) [file pbio.1001126.s009.pdf]

Supplementary Table 6. Median log2 magnitude of changes in expression between stages of spermatogenesis (2-fold cutoff)

2-fold cutoff	Early changes (premeiosis:meiosis)			Late changes (meiosis:postmeiosis)			Net change (premeiosis:postmeiosis)		
chromosomal arm	down	up	down - up	down	up	down - up	down	up	down - up
2L	-1.48	1.43	-0.05	-1.79	1.72	-0.07	-1.97	1.84	-0.13
2R	-1.41	1.41	0.00	-1.72	1.73	0.01	-2.05	1.74	-0.31
3L	-1.41	1.40	-0.01	-1.86	1.63	-0.24	-2.00	1.74	-0.26
3R	-1.40	1.40	0.00	-1.76	1.69	-0.07	-1.97	1.73	-0.24
4	-1.65	1.28	-0.37	-1.67	2.35	0.68	-1.63	2.16	0.53
X	-1.39	**1.26**	-0.13	**-1.64**	1.60	-0.04	**-1.81**	**1.70**	-0.11
A*	-1.41	**1.41**	0.00	**-1.78**	1.69	-0.09	**-2.00**	**1.75**	-0.25
X vs A (*MW P*-value)	0.433	**1.25E-09**		**0.009**	0.046		**1.63E-05**	0.281	

*autosomal totals exclude genes on the 4th chromosome
