# Supplementary material for: Sex Chromosome-Specific Regulation in the Drosophila Male Germline But Little Evidence for Chromosomal Dosage Compensation or Meiotic Inactivation
Source: PLoS Biol. 2011 Aug 16;9(8):e1001126. doi: 10.1371/journal.pbio.1001126 (PMC3156688; doi:10.1371/journal.pbio.1001126)
Supplement: Table S7 — RNAseq statistics. (PDF) [file pbio.1001126.s010.pdf]

Supplementary Table 7. RNAseq statistics

	wildtype testes	wildtype ovaries	*bam* testes	*bam* ovaries
total reads	30013546	29736261	32806238	41730579
mapped reads	19849063	22780977	20971625	21077212
# FlyBase transcripts (RPKM > 1)	11888	11256	12830	13077
# FlyBase genes (RPKM > 1)	9978	7811	8895	8963
